# Human induced pluripotent stem cell-derived neurons and coculture conditions regulate the adipogenic differentiation and functionality of human adipose stromal/stem cells

**DOI:** 10.1186/s12964-025-02544-x

**Published:** 2025-11-24

**Authors:** Sini Saarimaa, Miia Juntunen, Lotta Isosaari, Reija Autio, Marika Kuuskeri, Susanna Narkilahti, Susanna Miettinen

**Affiliations:** 1 Adult Stem Cell Group, Faculty of Medicine and Health Technology, Tampere University, Tampere, Finland; 2 NeuroGroup, Faculty of Medicine and Health Technology, Tampere University, Tampere, Finland; 3 Tays Research Services, Wellbeing Services County of Pirkanmaa, Tampere University Hospital, Tampere, Finland; 4 Health Sciences, Faculty of Social Sciences, Tampere University, Tampere, Finland; 5 Department of Plastic and Reconstructive Surgery, Center for Musculoskeletal Disease, Tampere University Hospital, Tampere, Finland

**Keywords:** Adipogenesis, Cellular communication, Combination medium, Human cells, Innervation, In vitro model, Lipolysis, Microphysiological systems, Neuro-adipose interactions, 3D cell culture

## Abstract

**Background:**

The obesity epidemic and associated diseases have increased the need to study human adipose tissue biology and, furthermore, the development of in vitro models of adipose tissues. Human adipose tissue innervation is a relatively understudied research area, and most studies have been performed in animal models. A common animal model is the mouse, which differs from humans in many areas, such as fat distribution, metabolism and genetics. Here, our aim was to develop a three-dimensional (3D) neuro-adipose in vitro model with human-derived cells to deepen the understanding of adipose tissue innervation. We hypothesized that our novel, optimized coculture conditions for neurons and adipose stromal/stem cells (ASCs) would enhance the adipogenesis of ASCs and the functionality of differentiating ASC-derived adipocytes.

**Methods:**

In this study, a novel 3D in vitro culture of adipocytes innervated on a microfluidic chip utilizing human ASCs and human induced pluripotent stem cell (hiPSC)-derived neurons was established. The cells were cultured in a fibrin–collagen 1 hydrogel in a microfluidic environment for a long period (≥ 21 days) in neuro-adipose combination medium (NM-AM). The adipogenic differentiation of ASCs and adipose cell functions, such as fatty acid (FA) uptake, lipolysis and adipokine secretion, were analyzed. In addition, cell activity was examined with calcium activity measurements, and cell connections were examined with immunocytochemistry and 3D confocal imaging.

**Results:**

The adipogenic differentiation of ASCs was significantly increased in NM-AM compared with adipogenic differentiation medium (AM) and differentiation was further enhanced by the neurons in the cocultures. Neurons formed synapses with each other as well as innervated ASCs. Both ASCs and neurons showed typical calcium activity in monocultures and cocultures. Neurons enhanced the FA uptake of ASCs while simultaneously decreasing the lipolysis of ASCs in cocultures. In addition, differentiating ASCs secreted adipokines and acetylcholine in mono- and cocultures.

**Conclusions:**

This research provides a novel human cell-based 3D in vitro model to study adipose tissue innervation and neuro-adipose interactions. Here, the novel human cell-based coculture model holds great potential for future mechanistic studies of neuro-adipose regulation.

**Supplementary Information:**

The online version contains supplementary material available at 10.1186/s12964-025-02544-x.

## Background

Adipose tissue, a metabolically active connective tissue, plays a primary role in energy homeostasis by regulating lipid metabolism, including the processes of lipogenesis, lipolysis and β-oxidation. Together, these processes allow adipocytes to manage the storage and release of energy as fatty acids (FAs) [1]. During lipogenesis, adipocytes store energy by taking up free FAs, which are esterified into triglycerides and packed into lipid droplets, whereas during lipolysis, triglycerides are hydrolyzed into glycerol and free FAs, and β-oxidation within mitochondria converts FAs into usable energy [1]. In addition to its energy-regulating functions, adipose tissue also serves as an endocrine organ, producing biologically active compounds that influence systemic metabolic processes, immunity, coagulation and angiogenesis [2]. As the global prevalence of obesity has nearly tripled over the past 30 years [3], obesity has become a pressing health issue, contributing to, e.g., cardiovascular diseases [4], type 2 diabetes [5, 6], certain cancers [7], and neurodegenerative diseases [8]. These findings underscore the need for research on adipose tissue regulation, especially the mechanisms underlying growth and metabolism, to advance both preventive and therapeutic strategies.

Like many tissues in the human body, adipose tissue is innervated [9, 10], and the central nervous system (CNS) regulates its functions via the peripheral nervous system (PNS), which consists of sympathetic and sensory neurons [11, 12]. Adipose tissue and the CNS are engaged in bidirectional communication: sympathetic neurons relay signals from the CNS to adipose tissue, whereas sensory neurons convey feedback from adipose tissue to the CNS [1, 11]. Nervous systems regulate adipogenesis through neural inputs, neuroendocrine signals and feedback mechanisms, adapting adipocyte development to the body’s metabolic state and energy demands [13, 14]. The sympathetic nervous system primarily inhibits adipogenesis via catecholamine release [14, 15], whereas hormones and neuropeptides such as neuropeptide Y can promote adipogenesis under various conditions [16]. Sympathetic activation is crucial for lipolysis, where adipocytes release FAs and hormones such as leptin as feedback [12, 14]. The role of the CNS in the development of obesity can be substantial, as it regulates many brain regions involved in energy homeostasis and food intake [17]. For example, chronic stress activates the hypothalamic–pituitary–adrenal axis, leading to increased cortisol levels, a hormonal response that can promote fat accumulation and adipogenesis [18].

Despite the critical role of neural input in adipose tissue functions, research on the innervation of human adipose tissue remains limited [10]. The absence of robust, innervated three-dimensional (3D) in vitro adipose tissue models has hindered the understanding of neuro-adipose interactions, which are essential for regulating metabolic processes and maintaining the functions of adipose tissue. While CNS pathways governing sympathetic outflow in obesity have been identified in preclinical animal studies [19–21], advanced in vitro models are needed to study these pathways in human cells. Most studies on adipose tissue innervation rely on rodent models in vivo [10, 22, 23] or in vitro [24]. However, human-specific organ-on-chip (OoC) models represent a promising alternative, allowing for human-relevant observations without interspecies differences [25–27]. Building an OoC model of innervated adipose tissue is challenging, as it requires a complex 3D environment, suitable media for different cell types, and extended culture periods to achieve cellular maturity and functionality.

* In vivo*, adipose tissue receives neuronal input via the PNS, which is regulated by centers within the CNS, including cortical regions involved in energy homeostasis and metabolic control [9–12]. Therefore, the use of human induced pluripotent stem cell (hiPSC)-derived cortical neurons (CNs) provides a defined and human-relevant neuronal population to model CNS-derived influences on adipose tissue. Although CNs do not directly innervate adipocytes in vivo, their inclusion enables the investigation of central neuronal modulation of adipogenesis and metabolism in a controlled in vitro setting, forming a first step toward more physiologically complex neuro-adipose models.

Here, we aim to address these research gaps by developing a 3D neuro-adipose OoC model in long-term (≥ 21 days) culture using human-derived cells on a microfluidic chip. In our model, the inclusion of human induced pluripotent stem cell (hiPSC)-derived cortical neurons (CNs) allows the study of CNS-like regulatory influences on differentiating adipose stromal/stem cells (ASCs) and their functions. This approach provides a novel way to examine how neuronal signals may impact adipocyte maturation and metabolic activity in vitro. Our findings indicate that a specialized neuro-adipose combination medium (NM-AM) enhances the adipogenic differentiation of ASCs and that coculture with CNs further promotes adipogenesis and FA uptake. Successfully sustaining functional CNs and ASCs in a 3D system within the proposed neuro-adipose model opens new avenues for studying the molecular impacts of obesity and supports the development of targeted therapeutic strategies.

## Methods

### Cell culture

#### Human adipose stromal/stem cells

Human ASCs were isolated as previously described [28] from subcutaneous adipose tissue samples, which were collected at the Tampere University Hospital Department of Plastic Surgery, from 3 donors (Supplementary Material 4, Table 1) who provided written and informed consent. The samples were collected and used under the supportive ethical statement of the Ethics Committee of the Expert Responsibility area of Tampere University Hospital (R15161). The ASCs were used between passages 2 and 5 and cultured in α-modified minimum essential medium (α-MEM, Gibco, USA) supplemented with 5% human serum (HS, Serana, Germany), 100 U/ml penicillin, and 100 µg/ml streptomycin (P/S, Euroclone, Italy) for 5 days in vitro (DIV) before plating for experiments. All the cultures were free of mycoplasma. The mesenchymal origin of the ASCs was confirmed with a surface marker expression analysis using flow cytometry (Supplementary Material 5, Table 2) and an assessment of their adipogenic, chondrogenic and osteogenic differentiation potential (Supplementary Material 6, Figure S1).

#### Human induced pluripotent stem cell-derived cortical neurons

The commercial mono-allelic mTagRFPT-tagged TUBA1B WTC hiPSC line (AICS-0031–035, Coriell Institute for Medical Research, USA) or in-house derived UTA.04511.WTs hiPSC line [29] was used for neuronal differentiation. The in-house-derived hiPSC line was derived at the iPS Cells facility, Faculty of Medicine and Health Technology (Tampere University, Finland) from donor who provided written informed consent. The Ethics Committee of the Expert Responsibility area of Tampere University Hospital has issued a supportive statement to use the hiPSC lines in research (R20159). The pluripotency of the hiPSC lines was monitored consistently, and all the cultures were free of mycoplasma and had preserved normal karyotypes. Both hiPSC lines were expanded and differentiated into CNs in feeder-free cultures according to previously published protocols [30, 31]. The differentiation protocol consisted of neural induction, neural proliferation, and maturation stages. The cells were cryopreserved on DIV 21 of differentiation. After thawing, the cells were differentiated until DIV 32 and plated for the final experiments. Differentiated CNs expressed typical neuronal markers, and microelectrode array (MEA) measurements were performed for the commercial TUBA1B WTC hiPSC line to ensure the development of spontaneous neuronal activity (Supplementary Material 7, Figure S2), as previously shown for UTA.04511.WTs-derived CNs [32].

### Formation of 3D cocultures on a microfluidic chip

**Fig. 1:**
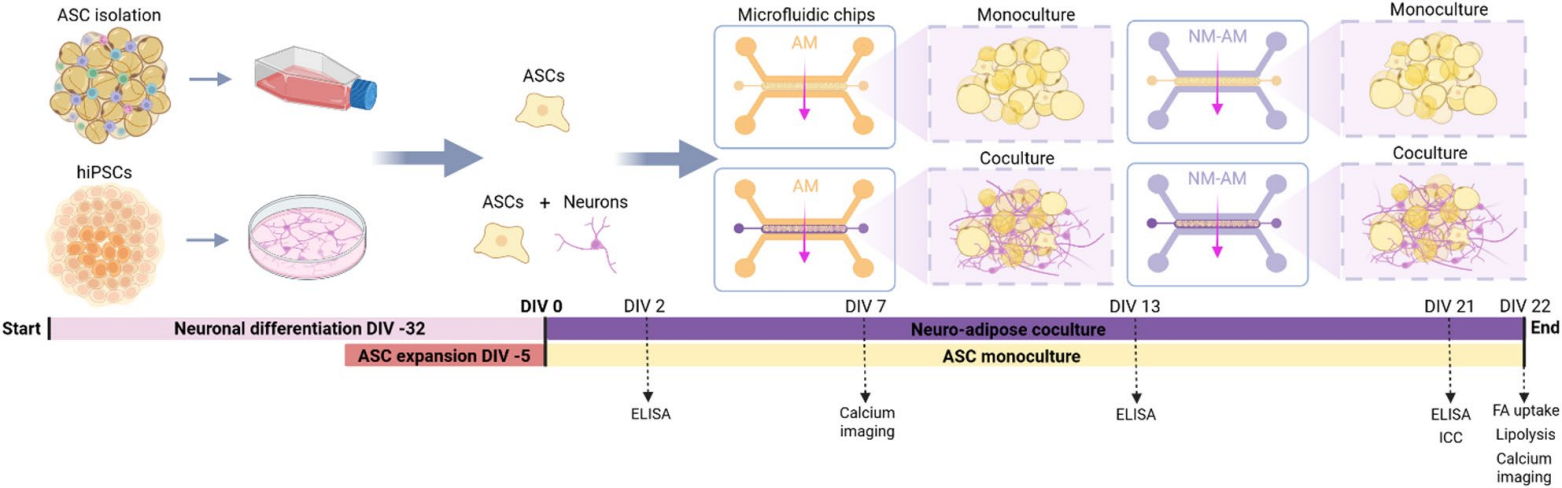
Experimental timeline for the establishment of 3D neuro-adipose cocultures on a microfluidic chip. Performed assays, timepoints and different culture conditions presented. Interstitial flow through the gels is indicated with pink arrows. Created in BioRender. Isosaari, L. (2025) https://BioRender.com/z45q349

The establishment of 3D cultures on a microfluidic chip (IdenTx 3, AIM Biotech, Singapore) was performed using a previously described protocol [33] for neurovascular cocultures, with slight modifications. Briefly, 3D neuro-adipose cocultures were formed on a microfluidic chip by adding ASCs and CNs to a hydrogel mixture consisting of 1.5 mg/ml human plasma fibrinogen (Sigma‒Aldrich, USA), 1 mg/ml rat tail collagen 1 (Gibco), and 0.6 iU/ml thrombin (Sigma‒Aldrich). The pH of the hydrogel solution was adjusted to 7 with 1 M sodium hydroxide (NaOH, Sigma‒Aldrich). ASCs were embedded at a density of 1.0 × 10^6^ cells/ml, and CNs were embedded at a density of 2.5 × 10^6^ cells/ml in 10 µl of hydrogel and immediately injected into the hydrogel channel of the chip (Fig. 1). DIV 0 refers to the start of the experiment hereafter.

Afterward, the cell-containing gels were allowed to polymerize inside the humidified chamber for 30–40 min in a + 37 °C cell culture incubator with 5% CO_2_ before the addition of cell culture medium. In total, 3 different media were used for cell culture. Neural maturation medium (NM) consisted of a 50:50 mixture of Dulbecco’s Modified Eagle Medium nutrient mixture F-12 (D-MEM/F12, with GlutaMAX, Gibco) and Neurobasal medium supplemented with 0.5% N2, 0.5% nonessential amino acids (NEAAs), 50 µM 2-mercaptoethanol, 1% B27 with retinoic acid, 0.5 mM GlutaMAX, 0.1% P/S (all from Thermo Fisher Scientific, USA), 2.5 µg/ml insulin (Sigma‒Aldrich), 10 ng/ml glial-derived neurotrophic factor (GDNF, R&D Systems, USA), 20 ng/ml brain-derived neurotrophic factor (BDNF, R&D Systems), 200 µM ascorbic acid (AA, Sigma‒Aldrich), and 500 µM dibutyryl-cyclic AMP (db-cAMP, Sigma‒Aldrich). A total of 10 µM Rho-kinase inhibitor (ROCKi, Sigma‒Aldrich) was added to the NM during cell seeding to support the survival of the cells. The adipogenic differentiation of ASCs was induced with adipogenic differentiation medium (AM), which was composed of α-MEM supplemented with 3% HS, 1% P/S, 100 nM insulin recombinant human zinc solution (Gibco), 1 µM dexamethasone, 33 µM biotin, and 17 µM D-pantothenate (all from Sigma‒Aldrich), or neuro-adipose combination medium (NM-AM), a 1:1 combination of AM and NM. During the first 7 days of culture, 1 µM rosiglitazone and 0.5 mM isobutylmethylxanthine (IBMX) (both from Sigma‒Aldrich) were added to AM to induce the adipogenic differentiation of ASCs. In addition, 40 µg/ml aprotinin (Abcam, USA) was added to all media during every medium change to prevent fibrin degradation and hydrogel shrinkage. The media were changed daily, and interstitial flow through the hydrogel was maintained by generating a gravity-based medium flow between the 2 medium channels of the chip by adding 90 µl of culture medium to both upper medium reservoirs and 50 µl of medium to both lower medium reservoirs. The chips were cultured inside a humidified incubator at + 37 °C with 5% CO_2_. Monocultures consisting only of ASCs or CNs were prepared and cultured at the same final cell densities (1.0 × 10^6^ cells/ml and 2.5 × 10^6^ cells/ml, respectively) in the same manner, and they acted as controls for the neuro-adipose cocultures. A summary of all the analyses performed, the cell lines used and the cell culture types with culture media are listed in the Supplementary Information (Supplementary Material 8, Table 4).

### Immunocytochemistry

On DIV 21, the 3D samples in the microfluidic chips were used for immunocytochemical (ICC) staining according to a previously published protocol [33]. Briefly, the samples were washed with 1× Dulbecco’s phosphate-buffered saline (DPBS, Euroclone) and fixed with 4% paraformaldehyde (PFA, Electron Microscopy Sciences, USA) for 30 min at room temperature (RT). Fixation was followed by 3 washes with 1× DPBS at RT. For permeabilization, the samples were treated with 0.1% Triton X-100 (Sigma‒Aldrich) in 1× DPBS for 10 min at RT and blocked with 0.1% Triton X-100, 1% bovine serum albumin (BSA, Sigma‒Aldrich), and 10% normal donkey serum (NDS, Millipore, Germany) in 1× DPBS for 2 h at RT. After blocking, the samples were incubated for 3 days at + 4 °C with the following primary antibodies diluted in 1× DPBS supplemented with 1% NDS, 0.1% Triton X-100, 1% BSA: anti-microtubule-associated protein 2 (MAP-2, chicken, NB300-213, 1:2000, Novus Biologicals, USA), anti-β-tubulin III (βtub_III_, chicken, ab41489, 1:100, Abcam), anti-synapsin 1 (synapsin-1, mouse, MA5-31919, 1:500, Invitrogen, USA), and anti-perilipin 1 (perilipin-1, rabbit, ab3526, 1:500, Abcam). Thereafter, the samples were washed 4 times with 1% BSA in 1× DPBS followed by an overnight incubation at + 4 °C with the following Alexa Fluor-labeled secondary antibodies diluted in 1× DPBS supplemented with 1% BSA: donkey anti-mouse 488 (A21202, 1:200), goat anti-chicken 488 (A11039, 1:200), goat anti-chicken 568 (A11041, 1:200), donkey anti-mouse 568 (A10037, 1:200) and donkey anti-rabbit 647 (A31573, 1:125, all from Thermo Fisher Scientific). In addition, 1 µg/ml of the nuclear marker 4’,6-Diamidino-2-phenylindole dihydrochloride (DAPI, 1:1500, Sigma‒Aldrich) and HCS LipidTOX green neutral lipid stain (1:1000, Invitrogen) were diluted in 1% BSA in 1× DPBS and added together with the secondary antibodies. The secondary antibody incubations were followed by 3 washes with 1× DPBS.

Fluorescence imaging was performed with a DMi8 inverted microscope (Leica Microsystems, Germany), and confocal imaging was performed with either a Nikon A1R superresolution microscope (Nikon Instruments, USA), an LSM 780 laser scanning confocal microscope with a Quasar spectral GaAsP detector (Carl Zeiss, Germany) or an Evident FLUOVIEW™ FV4000 laser scanning confocal microscope (Olympus, Germany). Images were processed with Fiji software [34], Imaris software (Oxford instruments, UK), and Huygens Essentials software (Scientific Volume Imaging, Netherlands). A 3D rendering of confocal images was created with Imaris software (Oxford instruments) to observe the innervation of differentiating adipocytes from different angles. ICC was repeated 2 times with ASC1 and ASC2 and 4 times with ASC3 donor cell line-derived ACSs, once with UTA.04511.WTs cell line-derived CNs and 7 times with TUBA1B WTC cell line-derived CNs.

### Surface area percentage of lipids in the differentiating ASCs

The surface area percentage of lipids in ASCs was determined on DIV 21 with the threshold method in Fiji software utilizing perilipin-1 and lipidTOX staining for ASC monocultures and cocultures in AM and NM-AM from 2D tile scan images throughout the microfluidic chip. A graph of the means ± standard deviations (SDs) was generated with GraphPad Prism 10.0.1 (GraphPad Software, USA). The assay was performed using 3 different ASC donor cell lines (ASC1, ASC2, and ASC3, *n* = 1 per cell line, total *n* = 3), together with the TUBA1B WTC-derived CNs in the cocultures.

### Fatty acid uptake analysis

Fatty acid (FA) uptake was analyzed for ASC mono- and cocultures in NM-AM medium on DIV 19 or 22, as previously described [35], with slight modifications. The fluorescently labeled medium chain FA analog 4,4-Difluoro-5-Methyl-4-Bora-3a,4a-Diaza-s-Indacene-3-Dodecanoic Acid (BODIPY, 500/510 C1, C12, Thermo Fisher Scientific) was added at 4 µM in the AM and applied to the samples similarly as medium. Fluorescence was imaged immediately for 60 min in 2 min sequences with a Leica DMi8 microscope under live-cell conditions. Time-lapse images were modified using Fiji software. Regions of interest (ROIs) containing single ASCs were manually selected and analyzed by assessing changes in the fluorescence intensity of the BODIPY label, which is definitive for FA uptake. Graphs of the changes in the mean intensity with 95% confidence intervals were generated with GraphPad Prism 10.0.1. The analysis was performed using 3 ASC donor cell lines (ASC1, ASC2 and ASC3, *n* = 10 per cell line, total *n* = 30), with the TUBA1B WTC-derived CNs used in the cocultures.

### Lipolysis assay

Lipolysis of differentiating ASCs in mono- and cocultures was performed on DIV 22. First, the chips were washed twice with 1× DPBS, followed by a lipolysis measurement before (BASAL) and after isoproterenol treatment (ISO) according to a previously published protocol [36], with slight modifications. For the basal lipolysis measurement, the chips were incubated in 2% FA-free BSA (Sigma‒Aldrich) in glucose- and phenol red-free Dulbecco’s Modified Eagle medium (DMEM, Gibco) for 3 h at + 37 °C. Thereafter, the medium from each gel of the chip was collected separately. Next, 2% FA-free BSA with 10 µM isoproterenol hydrochloride (Sigma‒Aldrich) in DMEM was added to the chips to measure β-adrenergic-induced lipolysis in ASCs, which were subsequently incubated at + 37 °C for 3 h, followed by media collection, as previously described. All the samples were stored at − 80 °C. The lipolysis assay was performed with a Glycerol-Glo Assay Kit (Promega, USA). The samples and standards were prepared according to the manufacturer’s instructions, and the luminescence was measured with a VICTOR Nivo multimode plate reader (PerkinElmer, USA). Median + MIN/MAX boxplots were generated with GraphPad Prism 10.0.1. The assay was performed using 3 different ASC donor cell lines (ASC1, ASC2, and ASC3, *n* = 3 per cell line, total *n* = 9), with the TUBA1B WTC-derived CNs used in the cocultures.

### Calcium imaging

Calcium imaging was performed on DIV 7 and 22 for CNs, ASCs, and cocultures in NM-AM, as previously described [33]. Briefly, chips were first washed with the extracellular solution (ECS) 3 times and then incubated in ECS supplemented with 5 µM FLUO4-AM (Abcam) or 5 µM FLUO8-AM (AAT Bioquest, USA) for 60 min at + 37 °C and subsequently washed twice with ECS, with a final wash for 30 min at + 37 °C. Immediately after the incubation, the chips were imaged with a Leica DMi8 system under live-cell imaging conditions (+ 37 °C, 5% CO_2_, 20% O_2_, 75% N_2_), and time-lapse videos were recorded up to 3 min with minimal time intervals. The time-lapse images were converted to grayscale (16-bit), and calcium oscillations were analyzed with Fiji software. Circular regions of somas were manually selected based on the differences in cell morphology between ASCs and CNs to separate the calcium signals from the different cell types. The selected cells were then analyzed as ROIs by assessing changes in the fluorescence intensity of the calcium indicator used. Graphs depicting the changes in fluorescence intensity of the ROIs were generated using GraphPad Prism 10.0.1. The variations in the average fluorescence intensity within the defined ROIs represented calcium oscillations. A semiautomated calcium oscillation analysis of the average amplitude and width of the peaks developed in house was performed using Fiji software and MATLAB R2019a v.9.6.0 software (MathWorks Inc., USA), as previously described [32]. The median + MIN/MAX boxplots of the average amplitude and peak width were generated with GraphPad Prism 10.0.1. Calcium imaging was performed with 3 ASC donor cell lines (ASC1, ASC2 and ASC3) and with TUBA1B WTC-derived CNs. The numbers of ROIs per group were as follows: ASC monoculture (*n* = 84), ASC coculture (*n* = 99), CN monoculture (*n* = 119), and CN coculture (*n* = 128).

### Enzyme-linked immunosorbent assay

Adiponectin and leptin secretion by differentiating ASCs in mono- and cocultures on DIV 2, 13 and 21 was assessed with Enzyme-Linked Immunosorbent Assays (ELISAs, R&D Systems) according to the manufacturer’s instructions. In addition, an ELISA of acetylcholine (Abcam) was performed on ASC and CN monocultures and cocultures at the same time points according to the instructions provided by the manufacturer. Conditioned medium samples were utilized for the analyses, and graphs of the means ± SDs were plotted with GraphPad Prism 10.0.1. Assays were performed with 3 different ASC donor cell lines (ASC1, ASC2, and ASC3) and 2 hiPSC-derived CN lines (UTA.04511.WTs and TUBA1B WTC). The numbers of collected samples were as follows: ASC monoculture in AM (*n* = 3) or NM-AM (*n* = 3–4), CN monoculture in NM-AM (*n* = 3–4), and coculture in NM-AM (*n* = 3–4), depending on the time point.

### Statistical analysis

All the statistical analyses were performed either with GraphPad Prism 10.0.1 or with IBM SPSS Statistics 29.0.1.0 (USA), whereas the nonparametric chi-square test of the surface area percentage of lipids in the ASCs was performed with MedCalc Software Ltd. 23.0.2 [37]. Linear regression analysis was used to compare the slopes of FA uptake by ASCs in mono- and coculture. The paired samples t test was used to assess lipolysis (BASAL versus ISO) and protein secretion (between different DIVs) in related samples. Additionally, one-way analysis of variance (ANOVA) was used for the same experiments while comparing the independent measurements across the culture conditions in terms of lipolysis and protein secretion. The nonparametric Mann‒Whitney U test was used to compare independent results of the calcium activity analysis from mono- and cocultures at DIV 22. When multiple statistical tests were conducted, the Bonferroni correction was applied where necessary. In cases where *n* ≤ 2, a statistical analysis was not performed. A *p* value < 0.05 was considered statistically significant (*p < 0.05, **p < 0.001,***p < 0.0001 and ****p < 0.00001).

### Data availability statement

 The datasets generated and analyzed during the current study are available from the corresponding author on reasonable request.

## Results

### NM-AM and neurons enhanced the adipogenic differentiation of ASCs

**Fig. 2:**
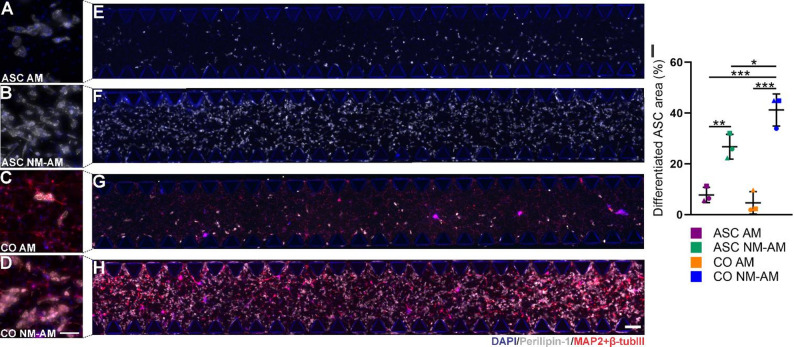
ICC staining of neuro-adipose cocultures and ASC monocultures on the chip at DIV 21.** A** ASC monoculture in AM. **B** ASC monoculture in NM-AM. **C** Coculture in AM. **D** Coculture in NM-AM. Tile scan images showing the event distribution of the cells throughout the microfluidic chip in the **E** ASC monoculture in AM, **F** ASC monoculture in NM-AM, **G** coculture in AM, and **H** coculture in NM-AM. The scale bars represent 300 μm. In **A**-**H**, perilipin-1 (gray) was used to stain proteins surrounding lipid droplets, while MAP-2 + βtub_III_ (red) costained neurons, and DAPI (blue) was used to label nuclei. **I** A 2D analysis of the area coverage of the differentiating ASCs in monocultures (ASC) and cocultures (CO) in AM or NM-AM. A nonparametric chi-square test was used, and the data are shown as the means ± SDs. The assay was repeated with 3 different ASC donor cell lines (*n* = 1 per cell line, total *n* = 3), which are represented by different patterns: ASC1 = circle, ASC2 = triangle and ASC3 = square. Significant differences are indicated as ^*^p < 0.05, ^**^p < 0.001 and ^***^p < 0.0001

The adipogenic differentiation of ASCs was evaluated by detecting perilipin-1 expression in collagen 1‒fibrin hydrogels in a microfluidic environment at DIV 21. Both ASCs and CNs were evenly distributed throughout the hydrogels in the chips in both the mono- and cocultures (Fig. 2A-H). NM-AM enhanced the 3D adipogenic differentiation of ASCs, as shown by the increased perilipin-1 expression (Fig. 2B and F), compared with AM alone (Fig. 2A and E). However, in all the cultures, the differentiating ASCs remained multilocular. The adipogenic differentiation of ASCs was further enhanced in cocultures with neurons cultured with NM-AM (Fig. 2D and H), whereas coculturing with AM did not increase differentiation (Fig. 2C and G). In addition, NM alone did not promote the adipogenic differentiation of ASCs in cocultures, and the CNs monocultured with NM or NM-AM neither expressed perilipin-1 nor were stained with LipidTOX (Supplementary Material 9, Figure S3). According to the 2D surface area analysis, both the mono- and cocultures in NM-AM contained significantly more differentiating ASCs than did the same cultures in AM (Fig. 2I, *p* = 0.0002 and *p* < 0.0001, respectively). In addition, in the NM-AM cocultures, neurons significantly increased adipogenic differentiation compared with that in the ASC monocultures in the NM-AM (Fig. 2I, *p* = 0.0442) and AM (Fig. 2I, *p* < 0.0001). These results suggest that NM-AM enhances the adipogenic differentiation of ASCs and that coculturing with neurons enhances differentiation even further.

### Successful long-term 3D neuro-adipose coculture on a chip

**Fig. 3:**
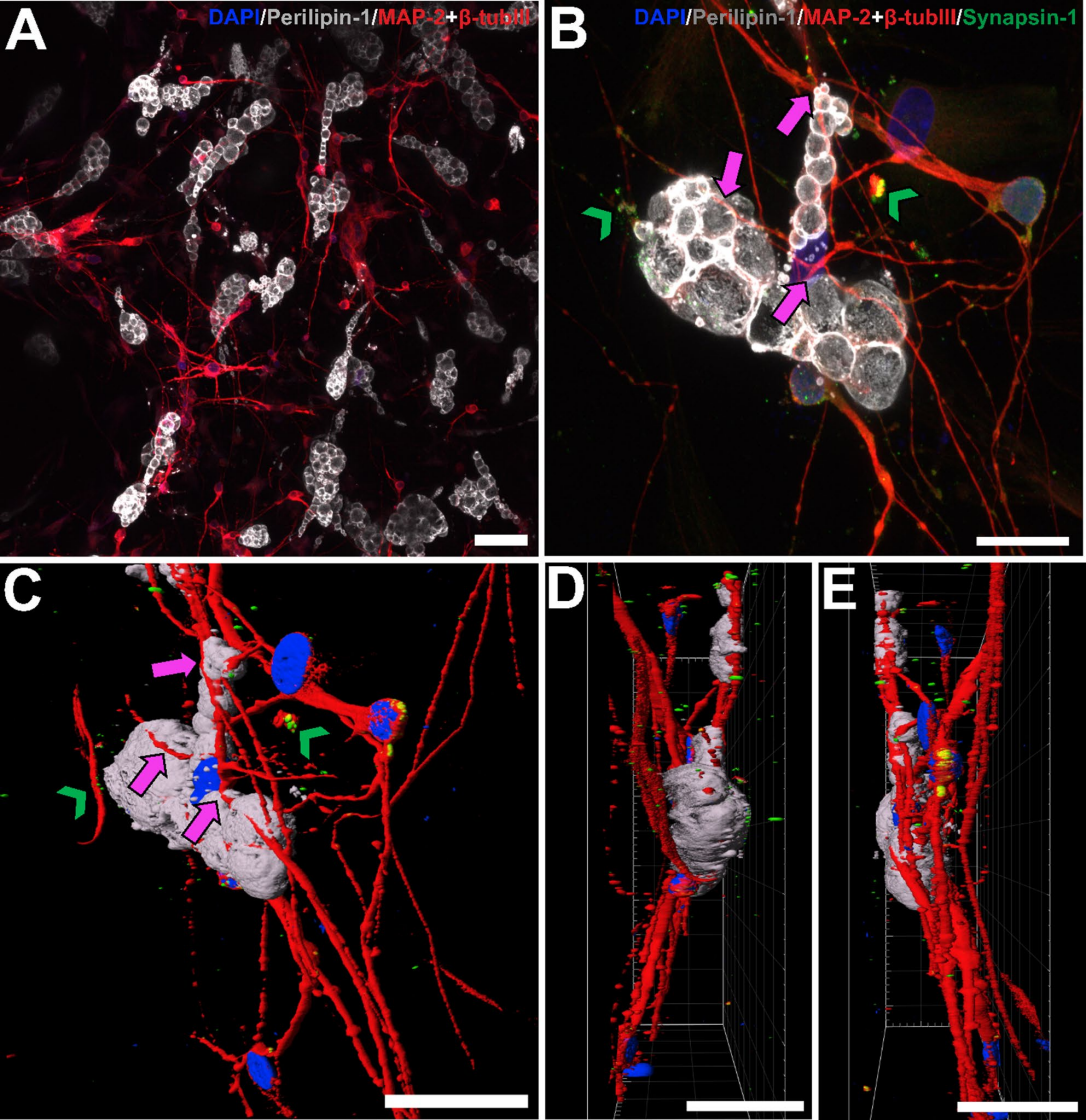
ICC staining of 3D neuro-adipose connections at DIV 21.** A** Staining for perilipin-1 (gray) showed ASCs undergoing adipogenic differentiation surrounded by neurons (MAP-2 + βtub_III_, red). **B** Neurites (MAP-2 + βtub_III_, red) formed connections with differentiating ASCs (perilipin-1, gray) and synaptic contacts (synapsin-1, green) with other neurons. The pink arrows indicate connections between neurons and differentiating ASCs, and the green arrowheads indicate synapses. **C** A 3D rendering of the image in **B**, where pink arrows indicate the neuro-adipose connections and green arrowheads indicate synapses shown as side views in **D-E**. The scale bars are 50 μm in each image

At DIV 21, ASCs cocultured with hiPSC-derived CNs formed physical interactions in NM-AM (Fig. 3). Differentiating adipocytes and neurons grew in close proximity to each other, enabling the formation of neuro-adipose connections (Fig. 3A). Neurites expressing MAP-2 + βtub_III_ wrapped around the maturing ASCs, suggesting successful innervation (Fig. 3B-E). Neurons also formed synapses expressing synapsin-1 with other neurons (Fig. 3B-E). With 3D rendering, the innervation of adipogenic differentiating ASCs was observed from different angles (Fig. 3C-E) (Supplementary Material 1, Supplementary Video 1).

### Neurons increased the FA uptake in cocultures, and NM-AM increased the lipolysis of differentiating ASCs

**Fig. 4:**
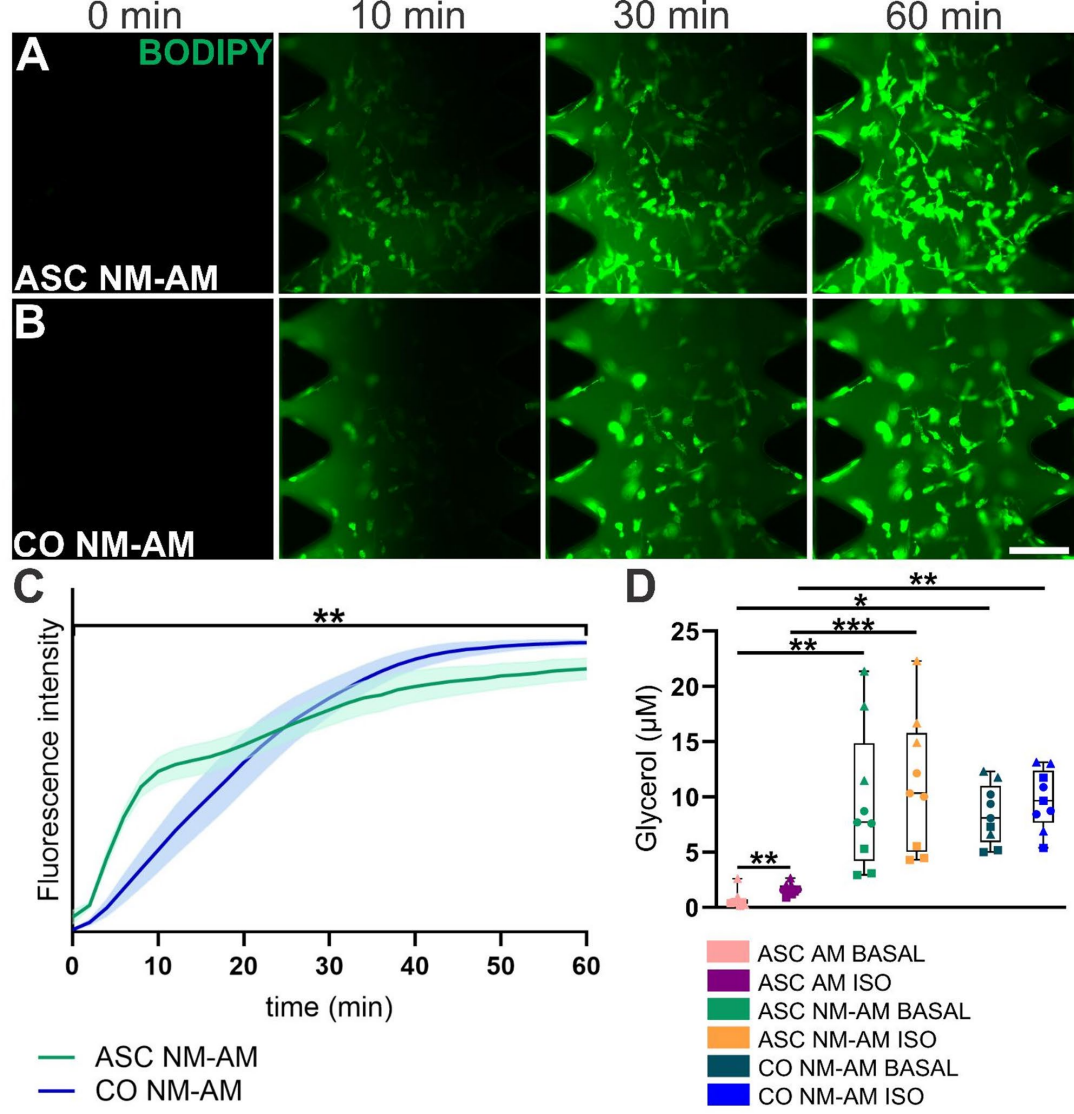
FA uptake and lipolysis of differentiating ASCs in mono- and cocultures.** A** Representative images of FA uptake by an ASC monoculture (ASC) in NM-AM and **B** images of a coculture (CO) in NM-AM. The fluorescence intensity of BODIPY (green) was measured from 0 to 60 min. Interstitial flow is shown from left to right. The scale bar is 300 μm. **C** Changes in fluorescence intensity (means) indicating FA uptake by ASCs in monoculture (green) and coculture (blue) along with the 95% confidence intervals. The assay was repeated with 3 ASC donor cell lines (ASC1, ASC2 and ASC3, *n* = 10 per cell line), an ASC monoculture (*n* = 30) and a coculture (*n* = 30). The difference in slopes between the cultures, determined with linear regression analysis, was statistically significant (*p* = 0.0002) across the entire duration of the analysis. **D** Lipolysis of differentiating ASCs in mono- and cocultures before (BASAL) and after isoproterenol (ISO) treatment in AM and NM-AM. Whiskers depict MIN/MAX, and horizontal lines show the median values. The assay was repeated with 3 different ASC donor cell lines (*n* = 3 per cell line, total *n* = 9): ASC1 = circle, ASC2 = triangle and ASC3 = square. Paired samples t tests and one-way ANOVA were used. Significant differences are indicated as ^*^ * *p* < 0.05, ^**^p < 0.001 and ^***^p < 0.0001

The free FA uptake of ASCs was monitored for 60 min in ASC mono- (Fig. 4A) and cocultures (Fig. 4B) in NM-AM following the introduction of BODIPY to cells with interstitial flow. ASCs were functionally active both in mono- and cocultures, as shown by the uptake of FAs. The uptake rate differed significantly between mono- and cocultures (Fig. 4C, *p* = 0.0002). FA uptake was faster in the ASC monocultures than in the cocultures in NM-AM during the first 28 min, after which FA uptake was higher in the cocultures until the end of the analysis (Fig. 4C).

A lipolysis assay was performed to study glycerol secretion by ASCs before (BASAL) and after treatment with the β-adrenergic agonist isoproterenol (ISO) in monocultures in AM and NM-AM and cocultures in NM-AM (Fig. 4D). In ASC monocultures in AM, ISO significantly increased the amount of glycerol released by ASCs compared to BASAL level (*p* = 0.00068). In NM-AM, glycerol secretion in the ASC monocultures and cocultures increased slightly after ISO exposure compared to BASAL secretion, but this difference did not reach statistical significance.

Furthermore, NM-AM significantly increased the lipolysis of ASCs at the BASAL level both in the ASC monoculture in NM-AM (*p* = 0.000277833) and in the coculture in NM-AM (*p* = 0.001347968) compared with the ASC monoculture in AM (Fig. 4D). Similarly, compared with the ASC AM culture, the NM-AM induced a significant increase in glycerol release after ISO treatment, both in the ASC monoculture (*p* = 0.0000627294) and coculture (*p* = 0.00046144) in NM-AM. However, neurons slightly decreased the lipolysis rate after ISO treatment in the NM-AM coculture compared with that in ASCs in the NM-AM monoculture, but the difference was not significant (Fig. 4D).

### Calcium activity changed during the maturation of ASCs and neurons and differed between mono- and cocultures

**Fig. 5:**
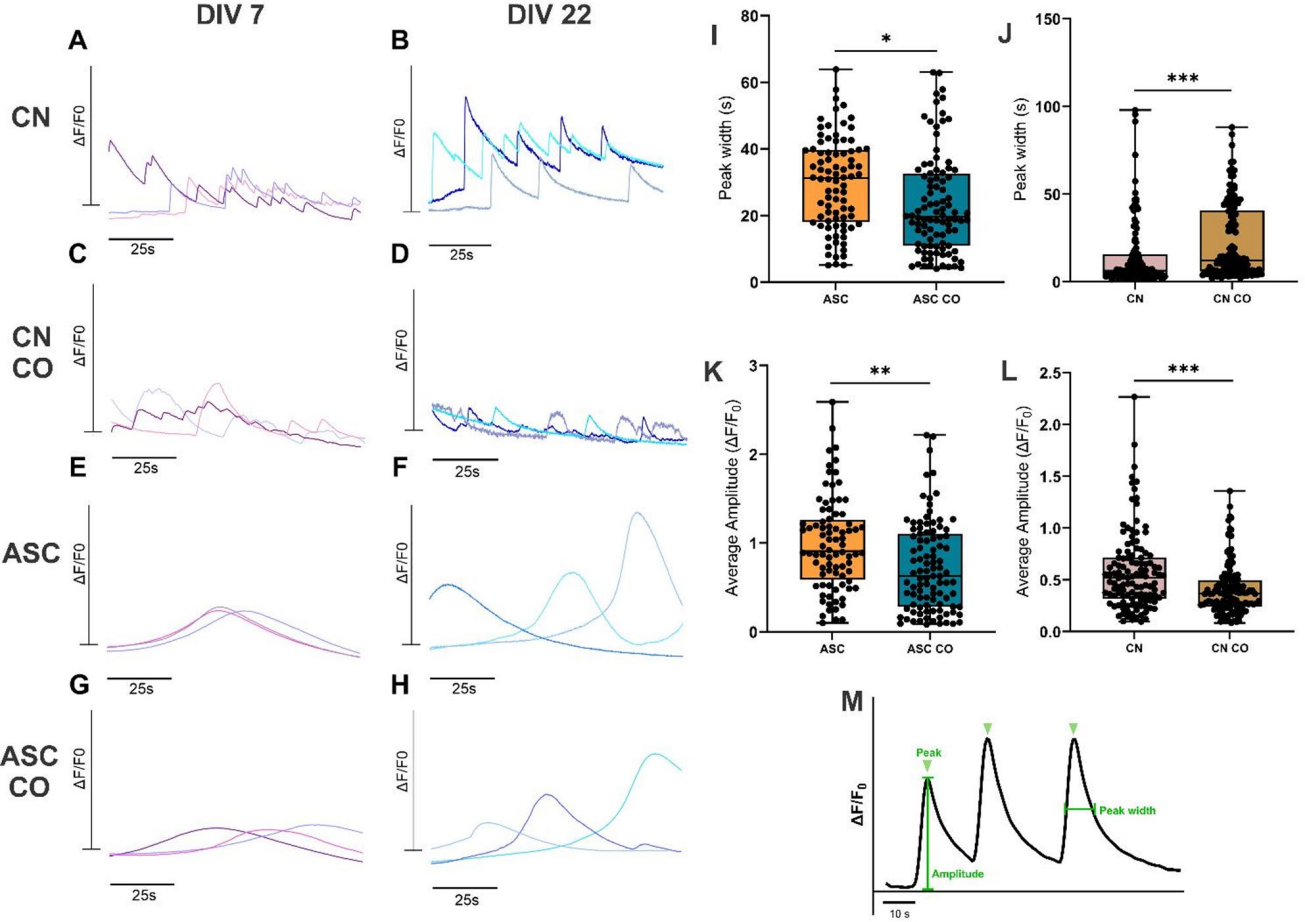
Calcium activity differed between mono- and cocultures. Representative calcium oscillation traces (*n* = 3) of CNs and differentiating ASCs in 3D cultures on microfluidic chips **A**-**H**. Changes in fluorescence intensity over the background (ΔF/F0) are shown with respect to time (100 s). **A**-**B** Calcium activity of CN monocultures (CN), **C**-**D** coculture CNs (CN CO), **E**-**F** ASC monocultures (ASC), and **G-H** coculture ASCs (ASC CO) in NM-AM on DIVs 7 and 22. Quantified parameters for the calcium peak dimension, showing the **I** peak width of ASCs and the **J** peak width of CNs in mono- and cocultures in NM-AM on DIV 22. **K** Average amplitude of ASCs and **L** average amplitude of CNs in mono- and cocultures in NM-AM on DIV 22. **M** The parameters derived from calcium oscillations (peak width and average amplitude) are displayed. Whiskers and horizontal lines indicate the ranges and median values, respectively, and each data point represents 1 measured cell. Calcium imaging was repeated with 3 ASC donor cell lines (ASC1, ASC2 and ASC3) and with TUBA1B WTC hiPSC line-derived CNs. The final number of cells per group was as follows: ASC (*n* = 84), ASC CO (*n* = 99), CN (*n* = 119) and CN CO (*n* = 128). The Mann‒Whitney U test was used, and significant differences are indicated as ^*^p < 0.05, ^**^p < 0.001 and ^***^p < 0.0001

The functionality of neurons and ASCs was studied with calcium imaging in monocultures (Supplementary Material 2-3, Supplementary Videos 2–3, respectively) and cocultures in NM-AM at DIV 7 and 22 (Fig. 5A-H). Active cells were detected under all culture conditions, and as the calcium waveforms of neurons and ASCs clearly differed from each other, cell-specific calcium signaling was also possible to identify under coculture conditions. The calcium waveforms in neurons were faster and spike-like (Fig. 5A-B), whereas those in differentiating ASCs were slower and slope-like (Fig. 5E-F). In addition, calcium oscillations occurred more frequently in neurons than in ASCs. Interestingly, the calcium activity of ASCs in mono- and cocultures increased during differentiation, and more calcium activity was detected on DIV 22 than on DIV 7 (5E-H). Neuronal calcium activity, on the other hand, increased temporally in monocultures (Fig. 5A-B), whereas it remained constant in cocultures (Fig. 5C-D). An in-depth semiautomated calcium oscillation analysis developed in house was performed for DIV 22 samples, with a focus on the peak width and amplitude (Fig. 5M). As shown in the representative images of the neuronal calcium waveforms (Fig. 5B, D), the signal amplitude was significantly higher in the CN monocultures than in the cocultures with ASCs (Fig. 5L, *p* = 0.000035), and the peak width was also significantly narrower in the monocultures than in the cocultures (Fig. 5J, *p* = 0.000044). Similarly, the signal amplitude in the ASC monocultures was significantly higher than that in the cocultures (Fig. 5K, *p* = 0.000625), whereas the peak width was narrower in the cocultures than in the monocultures (Fig. 5I, *p* = 0.001005). Taken together, both cell types expressed calcium-mediated activity, which, in the case of ASCs, seemed to evolve temporally despite the presence or absence of neurons. On the other hand, neurons seem to react to the presence of ASCs, as the typical temporal evolution in activity patterns did not occur in cocultures.

### Differentiating ASCs secreted adipokines and acetylcholine

**Fig. 6:**
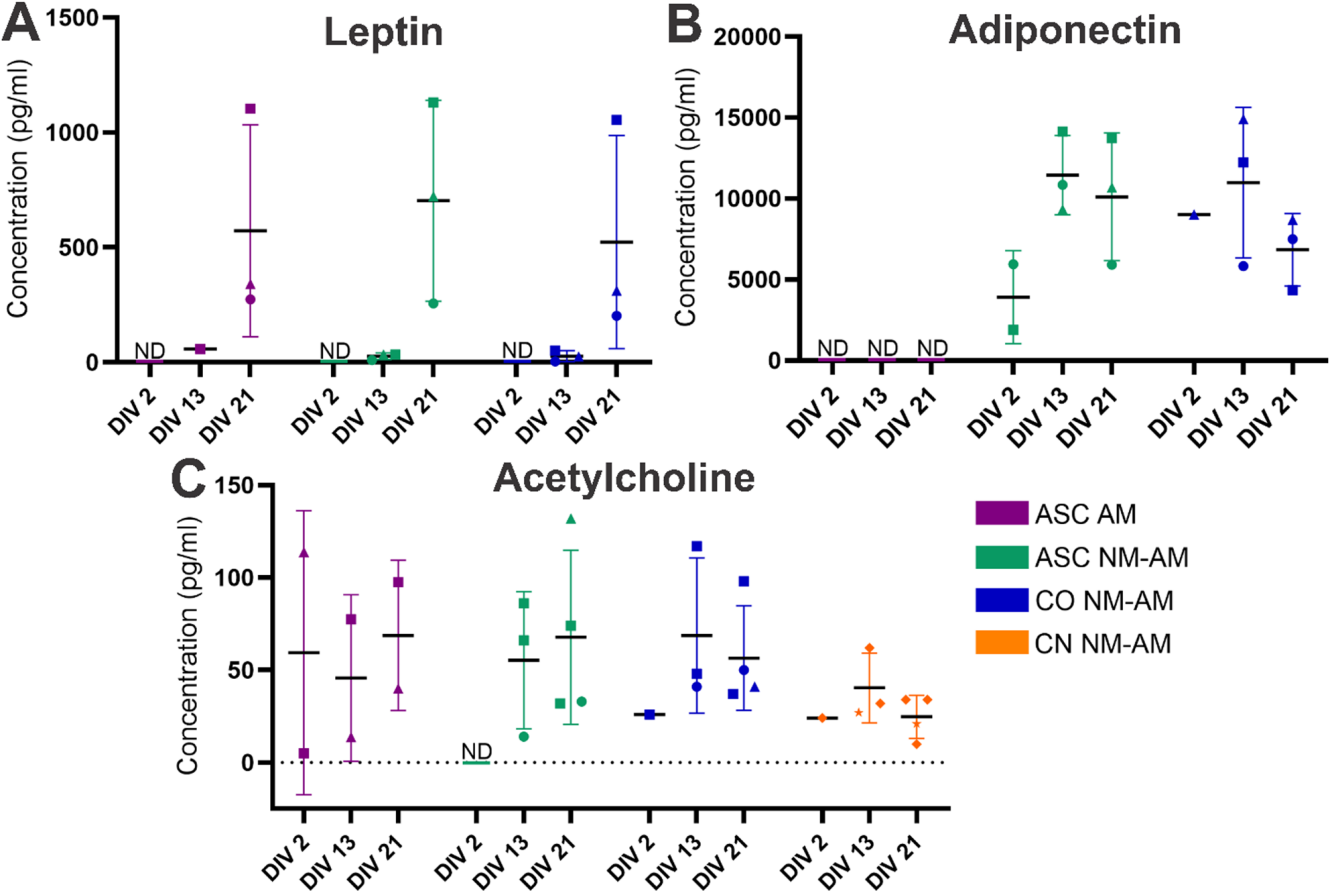
Differentiated ASCs secreted adipokines and acetylcholine. Leptin, adiponectin and acetylcholine secretion by ASCs and CNs at 2, 13 and 21 DIV in mono- and cocultures in AM and NM-AM. **A** Leptin secretion by ASC monocultures in AM (ASC AM) and NM-AM (ASC NM-AM) and cocultures in NM-AM (CO NM-AM) on DIV 2, 13 and 21. **B** Adiponectin secretion by ASC AM, ASC NM-AM and CO NM-AM on DIV 2, 13 and 21. **C** Acetylcholine secretion of ASC AM, ASC NM-AM, CO NM-AM and CN monocultures in NM-AM (CN NM-AM) on DIV 2, 13 and 21. The mean values and SDs are indicated with horizontal lines and whiskers, respectively. The assay was repeated with 3 different ASC donor cell lines: ASC1 = circle, ASC2 = triangle and ASC3 = square, with 2 hiPSC-derived CN lines; UTA.04511.WTs = star and TUBA1B WTC = diamond. The final number of measured cultures that yielded a result was as follows: ASC AM (*n* = 1–3), ASC NM-AM (*n* = 1–4), CN NM-AM (*n* = 1–4), and CO NM-AM (*n* = 1–4), depending on the time point. Paired samples t tests and one-way ANOVA were used, but statistical tests were not performed when *n* ≤ 2. ND indicates nondetectable values

Adipokine and acetylcholine secretion by ASCs and neurons under different culture conditions was assessed. Differentiating ASCs secreted leptin in both ASC monocultures in AM and NM-AM and in cocultures in NM-AM, with a temporal, nonsignificant, increase in secretion (Fig. 6A, *p* > 0.05). Adiponectin secretion was detected in both ASC monocultures and cocultures in NM-AM, but not in ASC monocultures in AM (Fig. 6B). Initially, in both ASC mono- and cocultures in NM-AM, adiponectin secretion increased over time but then started to decrease, although not significantly. Acetylcholine secretion was studied in the ASC monoculture in AM and in the CN monoculture, ASC monoculture and coculture in NM-AM (Fig. 6C). All cultures secreted acetylcholine at all time points except the ASC monoculture in NM-AM on DIV 2.

## Discussion

Adipose tissue and its interplay with the nervous system play critical roles in energy metabolism, hormonal regulation, and tissue homeostasis in the human body. To date, most neuro-adipose interaction studies have been performed in animal models in vivo [10, 23] or in vitro [24]; however, the translational relevance to human physiology remains limited due to interspecies differences. Some in vitro neuro-adipose studies have utilized human cells, where ASCs were cultured with fetal brain progenitor cells [38] or with hiPSC-derived peripheral neurons (PNs) [15]. Nevertheless, the culture periods have been relatively short (DIV 14–16) [15, 38] and have been conducted in 2D [15] or without an analysis of neuronal activity [38]. Our work addresses these limitations by creating a stable long-term (≥ 21 DIV) 3D neuro-adipose model with a fibrin–collagen type I hydrogel in a microfluidic chip that initiates the concurrent adipogenic differentiation of human ASCs and hiPSC-derived CNs innervation. Here, the extended culture time and physiologically relevant 3D microenvironment allowed the formation of cellular interactions, while the adipogenic differentiation and function of ASCs were significantly regulated by both NM-AM and innervation by hiPSC-derived neurons.

Medium selection plays an important role in cell differentiation and functionality in cocultures. Common adipogenic differentiation medium, similar to the AM used here, contains factors such as IBMX, dexamethasone, rosiglitazone and insulin [39, 40], which are known to promote the adipogenic differentiation of ASCs [41, 42]. Our novel combination of AM and NM significantly enhanced the adipogenesis of ASCs compared with either medium alone. One reason for this increase may be the higher concentration of insulin [40, 43], which was ~ 3X higher in the NM-AM than in the AM alone. Furthermore, NM contains AA and cAMP, which are not typically found in AM although they are known to support adipogenesis. AA promotes adipogenesis via increased collagen expression and synthesis [44] and cAMP-dependent signaling is crucial in the early stages of adipogenesis [45]. In previous neuro-adipose coculture studies, modified AM without D-pantothenate and biotin [38] or without serum, P/S, D-pantothenate and biotin combined with modified NM without P/S, GlutaMAX, 2-mercaptoethanol and NEAAs supplemented with neurotrophin-3, and nerve growth factor (NGF) [15] have been used. Our novel NM-AM contains all these components, except neurotrophin-3 and NGF, which constitute one explanatory factor underlying successful adipogenic differentiation.

In the human body, the CNS controls the functions of adipose tissue by innervating PNs [12]. Here, the cocultures showed consistent assemblies of neuro-adipose interactions across the hydrogel, with reproducible results observed between experiments. The expression of synapsin-1 indicated functional connections between neurons in both our CN mono- and cocultures, as suggested earlier in cocultures of human-derived PNs and mouse-derived ASCs [46]. Furthermore, the formation of physical connections was detected between neurons and adipogenic differentiating ASCs, as neurons surrounded the ASCs and wrapped around their surfaces, indicative of innervation under our coculture conditions. To the best of our knowledge, this study is the first to report neuro-adipose connections between ASCs undergoing adipogenic differentiation and hiPSC-derived CNs in 3D. A previous coculture study explored the interactions between human ASCs and human-derived PNs in 2D, with β-adrenergic receptor expression detected in ASCs [15]. Here, the synaptic marker expression and physical interactions between neurons and differentiating ASCs suggest functional interplay between these cells.

Neurons significantly increased the number of ASCs undergoing adipogenic differentiation, particularly in the NM-AM, based on the lipid surface area percentage analysis. Interestingly, Zhang and colleagues (2022) observed a negative effect of neural regulation on adipogenic differentiation based on expression of smaller lipid droplets in 3D bioprinted cocultures of human ASCs and human fetal brain progenitor cells compared with ASC monocultures [38]. In previous 2D coculture studies, adipogenesis in human ASCs was inhibited by human-derived sympathetic PNs [15], whereas it was enhanced by human-derived parasympathetic PNs [46]. These results suggest that human CNs can regulate adipogenesis by affecting lipid accumulation inside ASCs, either by decreasing [38] or increasing it, as shown in our study. Thus, the regulation of adipogenesis depends on the neuron type [15, 38, 46], and the medium composition used has an additional effect on adipogenesis, leading to the most abundant adipogenic differentiation of ASCs in NM-AM in the presence of CNs in our cocultures.

Calcium signaling activity serves as an indirect indicator of neuronal activity in vivo and in vitro [33, 47] and has been shown to support normal cellular functions in ASCs in vitro [48]. Calcium imaging was performed on both ASCs and neurons in mono- and cocultures in NM-AM to assess calcium signaling. Calcium activity was consistently observed across all culture conditions on DIVs 7 and 22. To our knowledge, this study is the first in which the calcium signaling of both ASCs and neurons was investigated in human cell-based cocultures. Throughout the culture period, calcium activity changed in both ASCs and neurons, with noticeable differences observed between mono- and cocultures. The oscillation patterns also vary between neurons and differentiating ASCs; neurons exhibit typical spike-like oscillations [33], whereas ASCs display oscillations similar to downward-opening parabolas. The spike-like oscillations in neurons, which were detected more frequently than those in ASCs, indicated faster calcium signaling dynamics characteristic of neurons [49]. These oscillations observed in CN monocultures aligned well with previously reported neuronal oscillation patterns in vitro [32, 33, 50]. Our findings indicate, however, that the coculture setup significantly influenced calcium signaling in neurons, notably reducing the amplitude of their oscillations compared with monocultures. Research has shown that the calcium oscillation frequency in ASCs is influenced by the lipid droplet size, with smaller lipid droplets yielding faster oscillations in mouse ASCs [51]. Additionally, ASCs are generally larger in size than are neurons [52], which may also influence the signaling differences. Coculturing also affected calcium signaling in ASCs, as monocultures presented significantly higher amplitudes and peak widths than cocultures did. A temporal increase in calcium activity in both the ASC mono- and cocultures was detected between DIV 7 and 22, corresponding to the progression of adipogenic differentiation. However, these findings contrast with previous findings, where calcium activity decreased during adipogenic differentiation in mouse ASCs [51] and human ASCs [53]. Neuronal activity was also noticeably increased in monocultures over time but was less pronounced in cocultures. The latter might be due to the suboptimal culture conditions for neurons in cocultures. In addition, neuronal calcium activity might be suppressed by the acetylcholine [54, 55] secreted by ASCs in our study. Since neuronal calcium oscillations correlate with cell maturity [49, 50], these findings suggest that neuro-adipose coculture may modulate calcium signaling dynamics and maturation in both ASCs and neurons relative to their monocultured counterparts.

During lipogenesis, free FAs are taken up by adipocytes, which is indicative of the functional activity of ASCs [56]. Here, a temporal increase in FA uptake by ASCs in cocultures compared with that in monocultures in NM-AM was observed, which is a novel finding suggesting a significant neuronal enhancement of the metabolic activity of ASCs. Previous studies have documented active FA metabolism in mature adipocytes in vitro [35, 57], but the effect of human-derived neurons on FA uptake in cocultures with human ASCs has not been previously reported. Lipolysis is an important metabolic process of adipocytes, where triglycerides are hydrolyzed into glycerol and free FAs when the need for energy increases [56]. Here, measurements following β-adrenergic isoproterenol stimulation revealed enhanced lipolysis across all culture conditions than under basal conditions. Previous studies have shown similar effects of β-adrenergic stimulation on human ASCs [36] and differentiated rat adipocyte monocultures [58]. Furthermore, lipolysis was increased in NM-AM monocultures compared to AM monocultures, possibly due to the improved adipogenic differentiation of ASCs in NM-AM. In previous coculture studies, both sympathetic [24] and parasympathetic neurons [46] were shown to downregulate β-adrenergic-stimulated lipolysis in mouse ASCs. Interestingly, lipolysis in our cocultures was slightly reduced, which is consistent with studies in animal-derived cocultures [24], suggesting a potential influence of neurons on the moderation of lipolytic activity.

Leptin and adiponectin are key indicators of adipocyte function and maturation and are the most abundant adipocytokines secreted by adipocytes [59–61]. Here, leptin secretion progressively increased during ASC differentiation, although it was slightly reduced in cocultures, mirroring findings from mouse-derived ASCs cocultured with rat-derived neurons [24]. In our study, adiponectin levels initially increased in NM-AM cocultures but declined after DIV 13 in both mono-and cocultures in NM-AM, with no apparent effect of neurons on adiponectin levels, in contrast with the findings of Zhang and colleagues (2022), who observed increased leptin secretion by human ASCs in 3D bioprinted cocultures with human neurons [38]. These findings suggest that adipokine modulation may depend on the neuronal subtype, culture medium, or cell source. Direct and indirect cholinergic signaling is crucial for adipose tissue functions and is utilized in crosstalk between the CNS and adipose tissue through acetylcholine [62]. Here, CN monocultures secreted acetylcholine at all time points, which is consistent with acetylcholine secretion in the CNS in vivo during cholinergic neurotransmission [63]. Moreover, acetylcholine secretion was observed in our ASC mono- and cocultures, regardless of the medium used. These results confirm the earlier results that ASCs cultured in vitro have the machinery to produce and secrete acetylcholine [64]. These secretion profiles of ASCs might regulate neuronal functionality [65] indicative of the interplay between these two cell types, which highlights the need for future studies. Taken together, our findings highlight that adipokine secretion and cholinergic signaling in ASCs are shaped by the culture conditions and cellular interactions with neurons.

## Conclusions

In summary, our successful long-term 3D neuro-adipose model provides new insights into neuro-adipose interactions, particularly the role of the CNS in enhancing ASC adipogenesis, metabolic activity, and functional signaling. Moreover, our model may reveal new molecules targeting adipose tissue through cell-cell interactions, potentially opening new avenues for drug discovery. A novel human cell-based coculture system was established with a commercially available microfluidic chip, and the innervation of differentiating adipocytes was observed. This research underscores the potential of our model for future mechanistic studies in neuro-adipose regulation, where optimized coculture conditions could yield mature adipocytes and allow for refined functional assessments.

Future work will focus on systematically evaluating the influence of neuronal and ASC densities, as well as the spatial proximity of neurons to differentiating adipocytes, to further elucidate how neural architecture shapes adipogenic outcomes. In addition, extending the model to include human PNs and mature adipocytes will enable closer physiological relevance, bridging central and peripheral aspects of neuro-adipose regulation and advancing toward a fully human innervated adipose tissue model.

## Supplementary Information

Supplementary Material 1. Supplementary Video 1: 3D rendering of adipogenic differentiating ASC innervation.

Supplementary Material 2. Supplementary Video 2: Calcium activity of neurons in monoculture.

Supplementary Material 3. Supplementary Video 3: Calcium activity of ASCs in monoculture.

Supplementary Material 4. Supplementary Table 1: Characteristics of the ASCs donors in the study.

Supplementary Material 5. Supplementary Table 2: Surface marker expression of ASCs. Supplementary Table 3: Antibodies for the surface marker expression analysis.

Supplementary Material 6. Supplementary Figure S1: Multilineage differentiation capacity of ASCs.

Supplementary Material 7. Supplementary Figure S2: Microelectrode array cultures, measurements and data analysis.

Supplementary Material 8. Supplementary Table 4: Performed analyses, used cell lines, cultures and media.

Supplementary Material 9. Supplementary Figure S3: ICC staining of neurons.

## Data Availability

The datasets used and/or analyzed during the current study are available from the corresponding author on reasonable request.

## Abbreviations

3D: Three-dimensional
α-MEM: α-Modified minimum essential medium
βtub: β-Tubulin III
AA: Ascorbic acid
ANOVA: Analysis of variance
ASC: Adipose stromal/stem cell
BDNF: Brain-derived neurotrophic factor
BODIPY: 4,4-Difluoro-5-Methyl-4-Bora-3a,4a-Diaza-s-Indacene-3-Dodecanoic Acid
BSA: Bovine serum albumin
CN: Cortical neuron
CNS: Central nervous system
CO: Coculture of neurons and ASCs
DAPI: Nuclear marker 4‘,6-diamidino-2-phenylindole
db-cAMP: Dibutyryl-cyclic AMP
D-MEM/F12: Dulbecco’s Modified Eagle Medium nutrient mixture F-12
DPBS: Dulbecco’s phosphate-buffered saline
ECS: Extracellular solution
ELISA: Enzyme linked immunosorbent assay
FA: Fatty acid
FABP4: Fatty acid binding protein 4
GDNF: Glial-derived neurotrophic factor
hiPSC: Human induced pluripotent stem cell
HS: Human serum
IBMX: Isobutylmethylxanthine
MAP-2: Microtubule-associated protein 2
MEA: Microelectrode array
Monoculture: Culture of neurons or culture of ASCs
NaOH: Sodium hydroxide
NDS: Normal donkey serum
NEAA: Nonessential amino acids
NGF: Nerve growth factor
NM: Neural maturation medium
NM-AM: Neuro-adipose combination medium
OoC: Organ-on-Chip
Perilipin-1: Anti-perilipin 1
PFA: Paraformaldehyde
PN: Peripheral neuron
PNS: Peripheral nervous system
P/S: Penicillin and streptomycin
ROCKi: Rho-kinase inhibitor
ROI: Region of interest
RT: Room temperature
SD: Standard deviation
Synapsin-1: Synapsin 1 monoclonal antibody

## References

[1] Zhu Q, Glazier BJ, Hinkel BC, Cao J, Liu L, Liang C, et al. Neuroendocrine regulation of energy metabolism involving different types of adipose tissues. Int J Mol Sci. 2019;20(11):2707. 31159462 10.3390/ijms20112707 PMC6600468

[2] Coelho M, Oliveira T, Fernandes R. Biochemistry of adipose tissue: an endocrine organ. Arch Med Sci AMS. 2013;9(2):191–200. 23671428 10.5114/aoms.2013.33181 PMC3648822

[3] Obesity. and overweight. https://www.who.int/news-room/fact-sheets/detail/obesity-and-overweight. Accessed 12 May 2025.

[4] Ashraf MJ, Baweja P. Obesity: the ‘Huge’ problem in cardiovascular diseases. Mo Med. 2013;110(6):499–504. 24564002 PMC6179812

[5] Ouchi N, Parker JL, Lugus JJ, Walsh K. Adipokines in inflammation and metabolic disease. Nat Rev Immunol. 2011;11(2):85–97. 21252989 10.1038/nri2921 PMC3518031

[6] Wang W, Zhu N, Yan T, Shi YN, Chen J, Zhang CJ, et al. The crosstalk: exosomes and lipid metabolism. Cell Commun Signal. 2020;18(1):119. 32746850 10.1186/s12964-020-00581-2 PMC7398059

[7] Guh DP, Zhang W, Bansback N, Amarsi Z, Birmingham CL, Anis AH. The incidence of co-morbidities related to obesity and overweight: a systematic review and meta-analysis. BMC Public Health. 2009;9:88. 19320986 10.1186/1471-2458-9-88 PMC2667420

[8] Neto A, Fernandes A, Barateiro A. The complex relationship between obesity and neurodegenerative diseases: an updated review. Front Cell Neurosci. 2023;17:1294420. 38026693 10.3389/fncel.2023.1294420 PMC10665538

[9] Bartness TJ, Ryu V. Neural control of white, beige and brown adipocytes. Int J Obes Suppl. 2015;5(1):S35–9. 27152173 10.1038/ijosup.2015.9 PMC4850578

[10] Wang Y, Leung VH, Zhang Y, Nudell VS, Loud M, Servin-Vences MR, et al. The role of somatosensory innervation of adipose tissues. Nature. 2022;609(7927):569–74. 36045288 10.1038/s41586-022-05137-7 PMC9477745

[11] Blaszkiewicz M, Willows JW, Johnson CP, Townsend KL. The importance of peripheral nerves in adipose tissue for the regulation of energy balance. Biology. 2019;8(1):10. 30759876 10.3390/biology8010010 PMC6466238

[12] Puente-Ruiz SC, Jais A. Reciprocal signaling between adipose tissue depots and the central nervous system. Front Cell Dev Biol. 2022;10:979251. 36200038 10.3389/fcell.2022.979251 PMC9529070

[13] Luo L, Liu M. Adipose tissue in control of metabolism. J Endocrinol. 2016;231(3):R77–99. 27935822 10.1530/JOE-16-0211 PMC7928204

[14] Bartness TJ, Liu Y, Shrestha YB, Ryu V. Neural innervation of white adipose tissue and the control of lipolysis. Front Neuroendocrinol. 2014;35(4):473–93. 24736043 10.1016/j.yfrne.2014.04.001 PMC4175185

[15] Fan Y, Huang S, Li F, Zhang X, Huang X, Li W, et al. Generation of functional and mature sympathetic neurons from human pluripotent stem cells via a neuroepithelial route. J Mol Neurosci. 2024;74(1):19. 38358571 10.1007/s12031-024-02196-5

[16] Kuo LE, Kitlinska JB, Tilan JU, Li L, Baker SB, Johnson MD, et al. Neuropeptide Y acts directly in the periphery on fat tissue and mediates stress-induced obesity and metabolic syndrome. Nat Med. 2007;13(7):803–11. 17603492 10.1038/nm1611

[17] Szalanczy AM, Key CCC, Woods LCS. Genetic variation in satiety signaling and hypothalamic inflammation: merging fields for the study of obesity. J Nutr Biochem. 2021;101:108928. 34936921 10.1016/j.jnutbio.2021.108928 PMC8959400

[18] Xiao Y, Liu D, Cline MA, Gilbert ER. Chronic stress, epigenetics, and adipose tissue metabolism in the obese state. Nutr Metab. 2020;17(1):88. 10.1186/s12986-020-00513-4 PMC7574417 33088334

[19] Head GA, Lim K, Barzel B, Burke SL, Davern PJ. Central nervous system dysfunction in obesity-induced hypertension. Curr Hypertens Rep. 2014;16(9):466. 25090962 10.1007/s11906-014-0466-4

[20] do Carmo JM, da Silva AA, Wang Z, Fang T, Aberdein N, de Lara Rodriguez CEP, et al. Obesity-induced hypertension: brain signaling pathways. Curr Hypertens Rep. 2016;18(7):58. 27262997 10.1007/s11906-016-0658-1 PMC5448788

[21] Guarino D, Nannipieri M, Iervasi G, Taddei S, Bruno RM. The role of the autonomic nervous system in the pathophysiology of obesity. Front Physiol. 2017;8:665. 28966594 10.3389/fphys.2017.00665 PMC5606212

[22] Chi J, Lin Z, Barr W, Crane A, Zhu XG, Cohen P. Early postnatal interactions between beige adipocytes and sympathetic neurites regulate innervation of subcutaneous fat. eLife. 2021;10:e64693. 33591269 10.7554/eLife.64693 PMC7990502

[23] Huesing C, Qualls-Creekmore E, Lee N, François M, Torres H, Zhang R, et al. Sympathetic innervation of inguinal white adipose tissue in the mouse. J Comp Neurol. 2021;529(7):1465–85. 32935348 10.1002/cne.25031 PMC7960575

[24] Turtzo LC, Marx R, Lane MD. Cross-talk between sympathetic neurons and adipocytes in coculture. Proc Natl Acad Sci U S A. 2001;98(22):12385–90. 11606782 10.1073/pnas.231478898 PMC60063

[25] Hagberg CE, Spalding KL. White adipocyte dysfunction and obesity-associated pathologies in humans. Nat Rev Mol Cell Biol. 2024;25(4):270–89. 38086922 10.1038/s41580-023-00680-1

[26] Vickers SP, Jackson HC, Cheetham SC. The utility of animal models to evaluate novel anti-obesity agents. Br J Pharmacol. 2011;164(4):1248–62. 21265828 10.1111/j.1476-5381.2011.01245.x PMC3229760

[27] Börgeson E, Boucher J, Hagberg CE. Of mice and men: pinpointing species differences in adipose tissue biology. Front Cell Dev Biol. 2022;10:1003118. 36187476 10.3389/fcell.2022.1003118 PMC9521710

[28] Kyllönen L, Haimi S, Mannerström B, Huhtala H, Rajala KM, Skottman H, et al. Effects of different serum conditions on osteogenic differentiation of human adipose stem cells in vitro. Stem Cell Res Ther. 2013;4(1):17. 23415114 10.1186/scrt165 PMC3706769

[29] Ojala M, Prajapati C, Pölönen RP, Rajala K, Pekkanen-Mattila M, Rasku J, et al. Mutation-Specific phenotypes in hiPSC-Derived cardiomyocytes carrying either Myosin-Binding protein C or α-Tropomyosin mutation for hypertrophic cardiomyopathy. Stem Cells Int. 2016;2016(1):1684792. 27057166 10.1155/2016/1684792 PMC4707351

[30] Hongisto H, Ilmarinen T, Vattulainen M, Mikhailova A, Skottman H. Xeno- and feeder-free differentiation of human pluripotent stem cells to two distinct ocular epithelial cell types using simple modifications of one method. Stem Cell Res Ther. 2017;8(1):291. 29284513 10.1186/s13287-017-0738-4 PMC5747074

[31] Hyvärinen T, Hyysalo A, Kapucu FE, Aarnos L, Vinogradov A, Eglen SJ, et al. Functional characterization of human pluripotent stem cell-derived cortical networks differentiated on laminin-521 substrate: comparison to rat cortical cultures. Sci Rep. 2019;9(1):17125. 31748598 10.1038/s41598-019-53647-8 PMC6868015

[32] Kapucu FE, Tujula I, Kulta O, Sukki L, Ryynänen T, Gram H, et al. Human tripartite cortical network model for Temporal assessment of alpha-synuclein aggregation and propagation in parkinson’s disease. Npj Park Dis. 2024;10(1):1–20. 10.1038/s41531-024-00750-x PMC11284226 39069518

[33] Isosaari L, Vuorenpää H, Yrjänäinen A, Kapucu FE, Kelloniemi M, Pakarinen TK, et al. Simultaneous induction of vasculature and neuronal network formation on a chip reveals a dynamic interrelationship between cell types. Cell Commun Signal. 2023;21(1):132. 37316873 10.1186/s12964-023-01159-4 PMC10265920

[34] Schindelin J, Arganda-Carreras I, Frise E, Kaynig V, Longair M, Pietzsch T, et al. Fiji: an open-source platform for biological-image analysis. Nat Methods. 2012;9(7):676–82. 22743772 10.1038/nmeth.2019 PMC3855844

[35] Rogal J, Binder C, Kromidas E, Roosz J, Probst C, Schneider S, et al. WAT-on-a-chip integrating human mature white adipocytes for mechanistic research and pharmaceutical applications. Sci Rep. 2020;10(1):6666. 32313039 10.1038/s41598-020-63710-4 PMC7170869

[36] Pieters VM, Rjaibi ST, Singh K, Li NT, Khan ST, Nunes SS, et al. A three-dimensional human adipocyte model of fatty acid-induced obesity. Biofabrication. 2022;14(4):045009. 10.1088/1758-5090/ac84b1 35896099

[37] Schoonjans F, MedCalc. May. MedCalc’s Comparison of proportions calculator. https://www.medcalc.org/calc/comparison_of_proportions.php. Accessed 22 2025.

[38] Zhang Y, Chen JW, Chen HY, Wang ZX, Li XD, Xu RX, et al. 3D bioprinted innervation ADMSC self-clustering culture model constructs for *in vitro* fat metabolism research: a preliminary study of ADMSC and neural progenitor cell co-culture model construct fabrication and characterization. Mater Today Chem. 2022;26:101092.

[39] Roxburgh J, Metcalfe AD, Martin YH. The effect of medium selection on adipose-derived stem cell expansion and differentiation: implications for application in regenerative medicine. Cytotechnology. 2016;68(4):957–67. 25795468 10.1007/s10616-015-9848-y PMC4960145

[40] Zebisch K, Voigt V, Wabitsch M, Brandsch M. Protocol for effective differentiation of 3T3-L1 cells to adipocytes. Anal Biochem. 2012;425(1):88–90. 22425542 10.1016/j.ab.2012.03.005

[41] Kim JY, Park EJ, Kim SM, Lee HJ. Optimization of adipogenic differentiation conditions for canine adipose-derived stem cells. J Vet Sci. 2021;22(4):e53. 34170094 10.4142/jvs.2021.22.e53 PMC8318799

[42] Hyväri L, Vanhatupa S, Halonen HT, Kääriäinen M, Miettinen S. Myocardin-related transcription factor A (MRTF-A) regulates the balance between adipogenesis and osteogenesis of human adipose stem cells. Stem Cells Int. 2020;2020(1):8853541. 33029150 10.1155/2020/8853541 PMC7527895

[43] Park A, Kim WK, Bae KH. Distinction of white, beige and brown adipocytes derived from mesenchymal stem cells. World J Stem Cells. 2014;6(1):33–42. 24567786 10.4252/wjsc.v6.i1.33 PMC3927012

[44] Liu C, Huang K, Li G, Wang P, Liu C, Guo C, et al. Ascorbic acid promotes 3T3-L1 cells adipogenesis by attenuating ERK signaling to upregulate the collagen VI. Nutr Metab. 2017;14(1):79. 10.1186/s12986-017-0234-y PMC5745638 29299041

[45] Petersen RK, Madsen L, Pedersen LM, Hallenborg P, Hagland H, Viste K, et al. Cyclic AMP (cAMP)-mediated stimulation of adipocyte differentiation requires the synergistic action of Epac- and cAMP-dependent protein kinase-dependent processes. Mol Cell Biol. 2008;28(11):3804–16. 18391018 10.1128/MCB.00709-07 PMC2423297

[46] Wu HF, Saito-Diaz K, Huang CW, McAlpine JL, Seo DE, Magruder DS, et al. Parasympathetic neurons derived from human pluripotent stem cells model human diseases and development. Cell Stem Cell. 2024;31(5):734-753.e8. 38608707 10.1016/j.stem.2024.03.011 PMC11069445

[47] Rosenberg SS, Spitzer NC. Calcium signaling in neuronal development. Cold Spring Harb Perspect Biol. 2011;3(10):a004259. 21730044 10.1101/cshperspect.a004259 PMC3179332

[48] Turovsky EA, Kaimachnikov NP, Turovskaya MV, Berezhnov AV, Dynnik VV, Zinchenko VP. Two mechanisms of calcium oscillations in adipocytes. Biochem Mosc Suppl Ser Membr Cell Biol. 2012;6(1):26–34.

[49] Johnson MA, Weick JP, Pearce RA, Zhang SC. Functional neural development from human embryonic stem cells: accelerated synaptic activity via astrocyte coculture. J Neurosci. 2007;27(12):3069–77. 17376968 10.1523/JNEUROSCI.4562-06.2007 PMC2735200

[50] Osaki T, Sivathanu V, Kamm RD. Engineered 3D vascular and neuronal networks in a microfluidic platform. Sci Rep. 2018;8(1):5168. 29581463 10.1038/s41598-018-23512-1 PMC5979969

[51] Turovsky EA, Turovskaya MV, Dynnik VV. Deregulation of Ca2+-signaling systems in white adipocytes, manifested as the loss of rhythmic activity, underlies the development of multiple hormonal resistance at obesity and type 2 diabetes. Int J Mol Sci. 2021;22(10):5109. 34065973 10.3390/ijms22105109 PMC8150837

[52] Hatton IA, Galbraith ED, Merleau NSC, Miettinen TP, Smith BM, Shander JA. The human cell count and size distribution. Proc Natl Acad Sci USA. 2023;120(39):e2303077120. 37722043 10.1073/pnas.2303077120 PMC10523466

[53] Torre EC, Bicer M, Cottrell GS, Widera D, Tamagnini F. Time-dependent reduction of calcium oscillations in adipose-derived stem cells differentiating towards adipogenic and osteogenic lineage. Biomolecules. 2021;11(10):1400. 34680033 10.3390/biom11101400 PMC8533133

[54] Rathouz MM, Vijayaraghavan S, Berg DK. Acetylcholine differentially affects intracellular calcium via nicotinic and muscarinic receptors on the same population of neurons ∗. J Biol Chem. 1995;270(24):14366–75. 7782297 10.1074/jbc.270.24.14366

[55] Picciotto MR, Higley MJ, Mineur YS. Acetylcholine as a neuromodulator: cholinergic signaling shapes nervous system function and behavior. Neuron. 2012;76(1):116–29. 23040810 10.1016/j.neuron.2012.08.036 PMC3466476

[56] Nguyen P, Leray V, Diez M, Serisier S, Bloc’h JL, Siliart B, et al. Liver lipid metabolism. J Anim Physiol Anim Nutr. 2008;92(3):272–83. 10.1111/j.1439-0396.2007.00752.x 18477307

[57] Louis F, Sowa Y, Kitano S, Matsusaki M. High-throughput drug screening models of mature adipose tissues which replicate the physiology of patients’ body mass index (BMI). Bioact Mater. 2022;7:227–41. 34466729 10.1016/j.bioactmat.2021.05.020 PMC8379425

[58] Louis C, Van den Daelen C, Tinant G, Bourez S, Thomé JP, Donnay I, et al. Efficient in vitro adipocyte model of long-term lipolysis: A tool to study the behavior of lipophilic compounds. Vitro Cell Dev Biol - Anim. 2014;50(6):507–18. 10.1007/s11626-014-9733-6 24477563

[59] Halvorsen YD, Bond A, Sen A, Franklin DM, Lea-Currie YR, Sujkowski D, et al. Thiazolidinediones and glucocorticoids synergistically induce differentiation of human adipose tissue stromal cells: biochemical, cellular, and molecular analysis. Metabolism. 2001;50(4):407–13. 11288034 10.1053/meta.2001.21690

[60] Lang K, Ratke J. Leptin and adiponectin: new players in the field of tumor cell and leukocyte migration. Cell Commun Signal. 2009;7(1):27. 20030801 10.1186/1478-811X-7-27 PMC2804604

[61] da Silva Rosa SC, Liu M, Sweeney G. Adiponectin synthesis, secretion and extravasation from circulation to interstitial space. Physiology. 2021;36(3):134–49. 33904786 10.1152/physiol.00031.2020 PMC8461789

[62] Shavva VS, Tarnawski L, Liu T, Ahmed O, Olofsson PS. Cholinergic signaling in adipose tissue. Curr Opin Endocr Metab Res. 2024;37:100546.

[63] Fadel JR. Regulation of cortical acetylcholine release: insights from in vivo microdialysis studies. Behav Brain Res. 2011;221(2):527–36. 20170686 10.1016/j.bbr.2010.02.022 PMC2925059

[64] El-Habta R, Kingham PJ, Backman LJ. Adipose stem cells enhance myoblast proliferation via acetylcholine and extracellular signal–regulated kinase 1/2 signaling. Muscle Nerve. 2018;57(2):305–11. 28686790 10.1002/mus.25741 PMC5811911

[65] Parimisetty A, Dorsemans AC, Awada R, Ravanan P, Diotel N, Lefebvre d’Hellencourt C. Secret talk between adipose tissue and central nervous system via secreted factors—an emerging frontier in the neurodegenerative research. J Neuroinflammation. 2016;13:67. 27012931 10.1186/s12974-016-0530-x PMC4806498

