# Supplementary material for: Human induced pluripotent stem cell-derived neurons and coculture conditions regulate the adipogenic differentiation and functionality of human adipose stromal/stem cells
Source: Cell Commun Signal. 2025 Nov 24;23:545. doi: 10.1186/s12964-025-02544-x (PMC12751193; doi:10.1186/s12964-025-02544-x)
Supplement: Supplementary file 4 — Supplementary Material 4. Supplementary Table 1: Characteristics of the ASCs donors in the study. [file 12964_2025_2544_MOESM4_ESM.docx]

**Supplementary Material 4**

**Supplementary Table 1.** Characteristics of the ASCs donors in the study

| **CELL LINE** | **AGE** | **SEX** | **BMI** | **TISSUE SOURCE** | **COLLECTION SITE** |
| --- | --- | --- | --- | --- | --- |
| DONOR1  ASC 1 | 48 | Female | 24.8 | Subcutaneous | Abdomen |
| DONOR2  ASC 2 | 57 | Female | 25.3 | Subcutaneous | Abdomen |
| DONOR3  ASC 3 | 56 | Female | 24.4 | Subcutaneous | Breast |
