# Supplementary material for: Human induced pluripotent stem cell-derived neurons and coculture conditions regulate the adipogenic differentiation and functionality of human adipose stromal/stem cells
Source: Cell Commun Signal. 2025 Nov 24;23:545. doi: 10.1186/s12964-025-02544-x (PMC12751193; doi:10.1186/s12964-025-02544-x)
Supplement: Supplementary file 5 — Supplementary Material 5. Supplementary Table 2: Surface marker expression of ASCs. Supplementary Table 3: Antibodies for the surface marker expression analysis. [file 12964_2025_2544_MOESM5_ESM.docx]

**Supplementary Material 5**

**Supplementary Table 2.** Surface marker expression of ASCs at passage 3 in the study

| **MARKER** | **ASC1** | **ASC2** | **ASC3** |
| --- | --- | --- | --- |
| **CD13** | 98.9 | 99.06 | 87.46 |
| **CD14** | 0.62 | 0.22 | 0.41 |
| **CD19** | 0.22 | 0.14 | 0.42 |
| **CD29** | 93.41 | 86.36 | 62.06 |
| **CD31** | 0.36 | 0.27 | 0.32 |
| **CD34** | 0.26 | 0.49 | 0.31 |
| **CD36** | 6.13 | 10.75 | 11.37 |
| **CD44** | 98.88 | 98.14 | 95.43 |
| **CD45RO** | 0.82 | 0.39 | 0.57 |
| **CD54** | 4.17 | 4.26 | 0.79 |
| **CD73** | 99.92 | 99.97 | 99.96 |
| **CD90** | 99.89 | 99.75 | 99.94 |
| **CD105** | 99.73 | 99.09 | 98.83 |
| **CD146** | 4.02 | 0.6 | 0.49 |
| **CD235A** | 0.32 | 0.37 | 0.31 |
| **HLA-DR** | 0.37 | 0.31 | 0.47 |

**Surface marker expression analysis**

The surface marker expression of ASCs was assessed at passage 3 to verify the phenotypic characteristics of the ASCs according to the International Society for Cell and Gene Therapy (ISCT) [1] and the International Federation for Adipose Therapeutics and Science (IFATS) [2]. Antibodies against CD13-BV421, CD14-APC, CD19-APC, CD29-FITC, CD31-BV421, CD34-APC, CD36-APC, CD44-FITC, CD45RO-APC, CD54-FITC, CD73-PE, CD90-APC, CD105-PE, CD146-BV421, CD235A-BV421 and HLA-DR BV421 (Supplementary Table 3) were used. ASCs were analyzed with a CytoFlex S flow cytometer (Beckman Coulter). A total of 10000 events were recorded during the flow cytometry analysis, and the percentage of each positive cell was determined.

**Supplementary Table 3.** Antibodies for the surface marker expression analysis

| **ANTIBODIES** | **SOURCE** | **IDENTIFIER** |
| --- | --- | --- |
| BV421 Mouse Anti-Human CD13 | BD Biosciences | 562596 |
| APC Mouse Anti-Human CD14 | BD Biosciences | 561708 |
| APC Mouse Anti-Human CD19 | BD Biosciences | 561742 |
| Anti human CD29 MEM-101A IgG1 FITC | Immunotools | 21270293 |
| BV421 Mouse Anti-Human CD31 | BD Biosciences | 564089 |
| APC- conjugated monoclonal antibody to human CD34 | Immunotools | 21270346 |
| APC Mouse Anti-Human CD36. Clone CB38 | BD Pharmingen | 561822 |
| FITC Mouse Anti-Human CD44 | BD Biosciences | 560977 |
| CD45RO-APC | BD Biosciences | 340438 |
| Human ICAM-1/CD54 Fluorescein-conjugated Antibody | R&D Systems | BBA20 |
| FITC Mouse Anti-Human CD73 | BD Biosciences | 561254 |
| APC Mouse Anti-Human CD90 | BD Pharmingen | 561971 |
| FITC Mouse anti-Human CD105 | BD Biosciences | 561443 |
| BV421 Mouse Anti-Human CD146 | BD Biosciences | 566226 |
| BV421 Mouse Anti-Human CD235a | BD Biosciences | 562938 |
| BV421 Mouse anti-human HLA-DR | BD Biosciences | 562804 |

**References**

1. Dominici M, Le Blanc K, Mueller I, Slaper-Cortenbach I, Marini F, Krause D, et al. Minimal criteria for defining multipotent mesenchymal stromal cells. The International Society for Cellular Therapy position statement. Cytotherapy. 2006;8(4):315-7.

2. Bourin P, Bunnell BA, Casteilla L, Dominici M, Katz AJ, March KL, et al. Stromal cells from the adipose tissue-derived stromal vascular fraction and culture expanded adipose tissue-derived stromal/stem cells: a joint statement of the International Federation for Adipose Therapeutics and Science (IFATS) and the International Society for Cellular Therapy (ISCT). Cytotherapy. 2013;15(6):641-8.
