# Supplementary material for: Human induced pluripotent stem cell-derived neurons and coculture conditions regulate the adipogenic differentiation and functionality of human adipose stromal/stem cells
Source: Cell Commun Signal. 2025 Nov 24;23:545. doi: 10.1186/s12964-025-02544-x (PMC12751193; doi:10.1186/s12964-025-02544-x)
Supplement: Supplementary file 6 — Supplementary Material 6. Supplementary Figure S1: Multilineage differentiation capacity of ASCs. [file 12964_2025_2544_MOESM6_ESM.docx]

**Supplementary Material 6**

**Multilineage differentiation capacity of ASCs**

**Osteogenic differentiation**

The osteogenic differentiation potential of ASCs was assessed at passage 3 or 4. ASCs were plated as previously described [1] and differentiated in osteogenic medium for 21 or 26 DIV. Alizarin Red staining was performed as previously described [2] to study the potential of the cells to produce mineralized calcium deposits. The cells were photographed with a Canon Digital IXUS 100IS or Canon EOS M50 mark II camera.

**Chondrogenic differentiation**

The chondrogenic differentiation potential of ASCs was assessed at passages 4 or 5. ASCs were plated as micro masses as previously described [3] and differentiated toward chondrocytes for 14 DIV. After differentiation, Alcian blue staining was performed as previously described [3] to observe the sulfated glycosaminoglycans of differentiated cells. Stained histological sections were photographed with a Hamamatsu S60 microscope scanner.

**Adipogenic differentiation**

The adipogenic differentiation potential of ASCs was assessed at passage 3 or 4. The cells were plated into a 24-well plate as previously described [4], [5]. ASCs were differentiated in adipogenic medium for 21 DIV. Lipid accumulation was subsequently observed with Oil Red O staining, as previously described [6]. The cells were photographed with a phase contrast microscope Nikon Eclipse TE 2000-S (Japan) and Nikon Digital Sight DS-L1 camera (Japan).


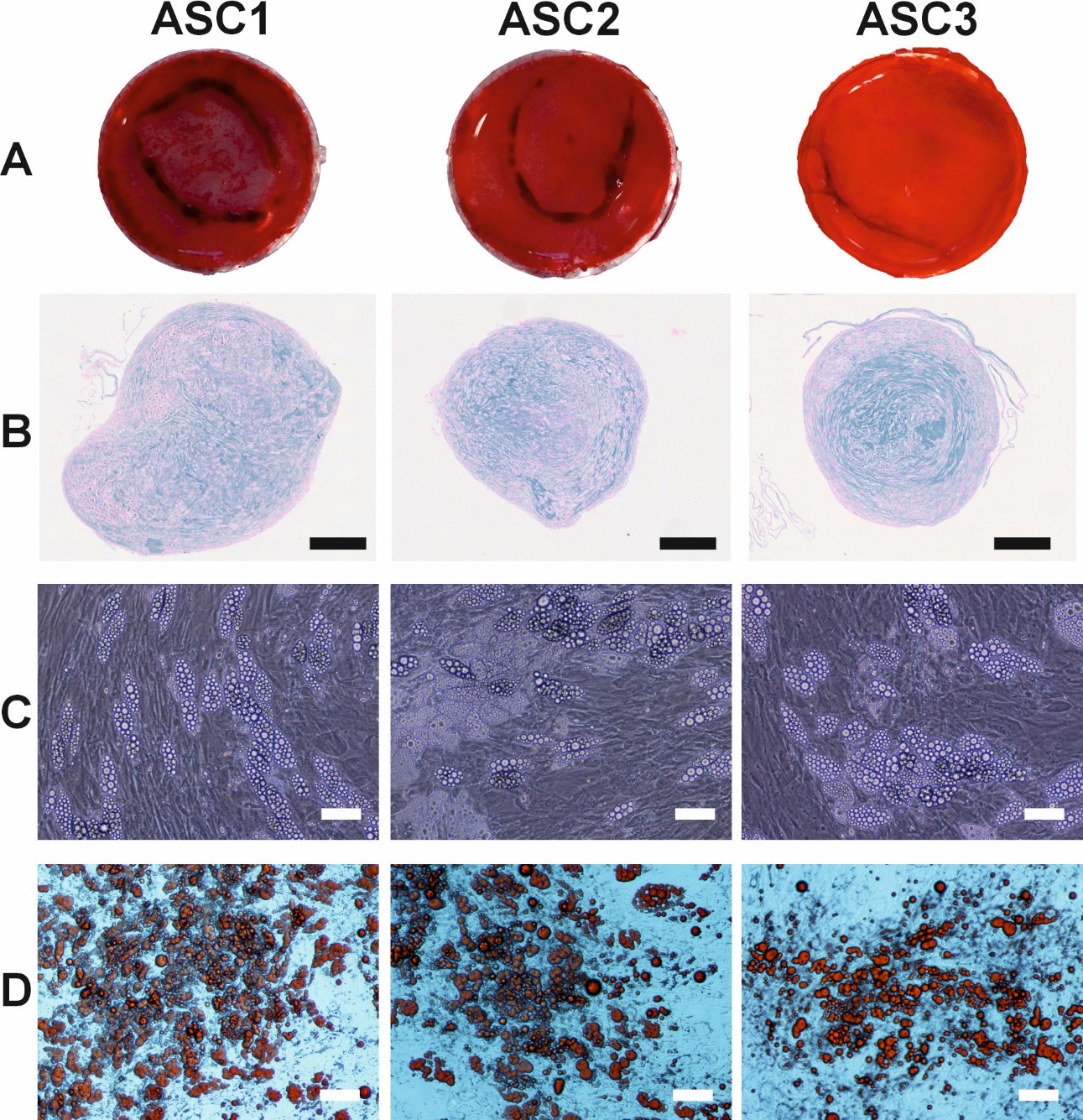


**Supplementary Figure S1.** Multipotential differentiation capacity of ASCs. **A** Osteogenic differentiation; Alizarin Red. **B** Chondrogenic differentiation; Alcian blue. Scale bar 200 µm. **C** Adipogenic differentiation; phase contrast images after 21 DIV and **D** after Oil Red O staining (red). Scale bar 100 µm.

**References**

1. Hyväri L, Ojansivu M, Juntunen M, Kartasalo K, Miettinen S, Vanhatupa S. Focal adhesion kinase and ROCK signaling are switch-like regulators of human adipose stem cell differentiation towards osteogenic and adipogenic lineages. Stem Cells International. 2018;2018:2190657.

2. Ojansivu M, Vanhatupa S, Björkvik L, Häkkänen H, Kellomäki M, Autio R, et al. Bioactive glass ions as strong enhancers of osteogenic differentiation in human adipose stem cells. Acta Biomaterialia. 2015;21:190-203.

3. Juntunen M, Heinonen S, Huhtala H, Rissanen A, Kaprio J, Kuismanen K, et al. Evaluation of the effect of donor weight on adipose stromal/stem cell characteristics by using weight-discordant monozygotic twin pairs. Stem Cell Research & Therapy. 2021;12(1):516.

4. Herbers E, Patrikoski M, Wagner A, Jokinen R, Hassinen A, Heinonen S, et al. Preventing white adipocyte browning during differentiation *in vitro*: The effect of differentiation protocols on metabolic and mitochondrial phenotypes. Stem Cells International. 2022;2022:3308194.

5. Mahmoud M, Juntunen M, Adnan A, Kummola L, Junttila IS, Kelloniemi M, et al. Immunomodulatory functions of adipose mesenchymal stromal/stem cell derived from donors with type 2 diabetes and obesity on CD4 T cells. Stem Cells. 2023;41(5):505-519.

6. Patrikoski M, Juntunen M, Boucher S, Campbell A, Vemuri MC, Mannerström B, et al. Development of fully defined xeno-free culture system for the preparation and propagation of cell therapy-compliant human adipose stem cells. Stem Cell Research & Therapy. 2013;4(2):27.
