# Supplementary material for: Human induced pluripotent stem cell-derived neurons and coculture conditions regulate the adipogenic differentiation and functionality of human adipose stromal/stem cells
Source: Cell Commun Signal. 2025 Nov 24;23:545. doi: 10.1186/s12964-025-02544-x (PMC12751193; doi:10.1186/s12964-025-02544-x)
Supplement: Supplementary file 7 — Supplementary Material 7. Supplementary Figure S2: Microelectrode array cultures, measurements and data analysis. [file 12964_2025_2544_MOESM7_ESM.docx]

**Supplementary Material 7**

**Microelectrode array cultures, measurements and data analysis**

On DIV 32 of neuronal differentiation, human induced pluripotent stem cell (hiPSC)-derived cortical neurons (CNs) from the commercial TUBA1B WTC hiPSC line (AICS-0031-035, Coriell Institute for Medical Research) were detached and seeded onto Axion CytoView MEA 48 plates (Axion BioSystems, USA) to characterize the functional development of the differentiated neurons, as previously described [1]. Briefly, MEA plates were coated with 0.1% polyethyleneimine (PEI, Sigma‒Aldrich) and 50 mg/ml human recombinant laminin-521 (LN521, BioLamina, Sweden), and the neurons were seeded on the coated plates at a density of 635 000 cells/cm^2^ in NM supplemented with 10 µM ROCKi. After 1 DIV, the medium was changed to a 1:1 mixture of NM and Brainphys maturation medium (BMM), which included Brainphys neuronal medium (Stemcell Technologies), 1% N2, 1% B27, 0.1% penicillin/streptomycin, 20 ng/ml BDNF, 10 ng/ml GDNF, 500 μM db-cAMP and 200 μM AA. Two days after seeding, the medium was changed to 100% BMM, after which the cells were maintained in the BMM, and the medium was changed 3 times a week. MEA recordings of the neuronal networks were conducted 2 times a week for 10 min until 37 DIV on MEA (DOM) with the Axion Maestro system controlled by AxIS software (Axion Biosystems, USA) using a 12.5 kHz sampling rate at 37 °C, as previously described [1]. As reported in a previous study [1], the analysis of MEA data consisted of spike detection with an in-house-generated MATLAB (MathWorks) script and further processing of the spike data with meaRtools [2] to obtain the MEA parameters. Supplementary Figure S2 shows the typical expression of the neuronal markers MAP-2+βtub_III_ and the development of spontaneous neuronal network activity, along with both the firing rate (Supplementary Figure S2B) and burst rate (Supplementary Figure S2C) parameters.


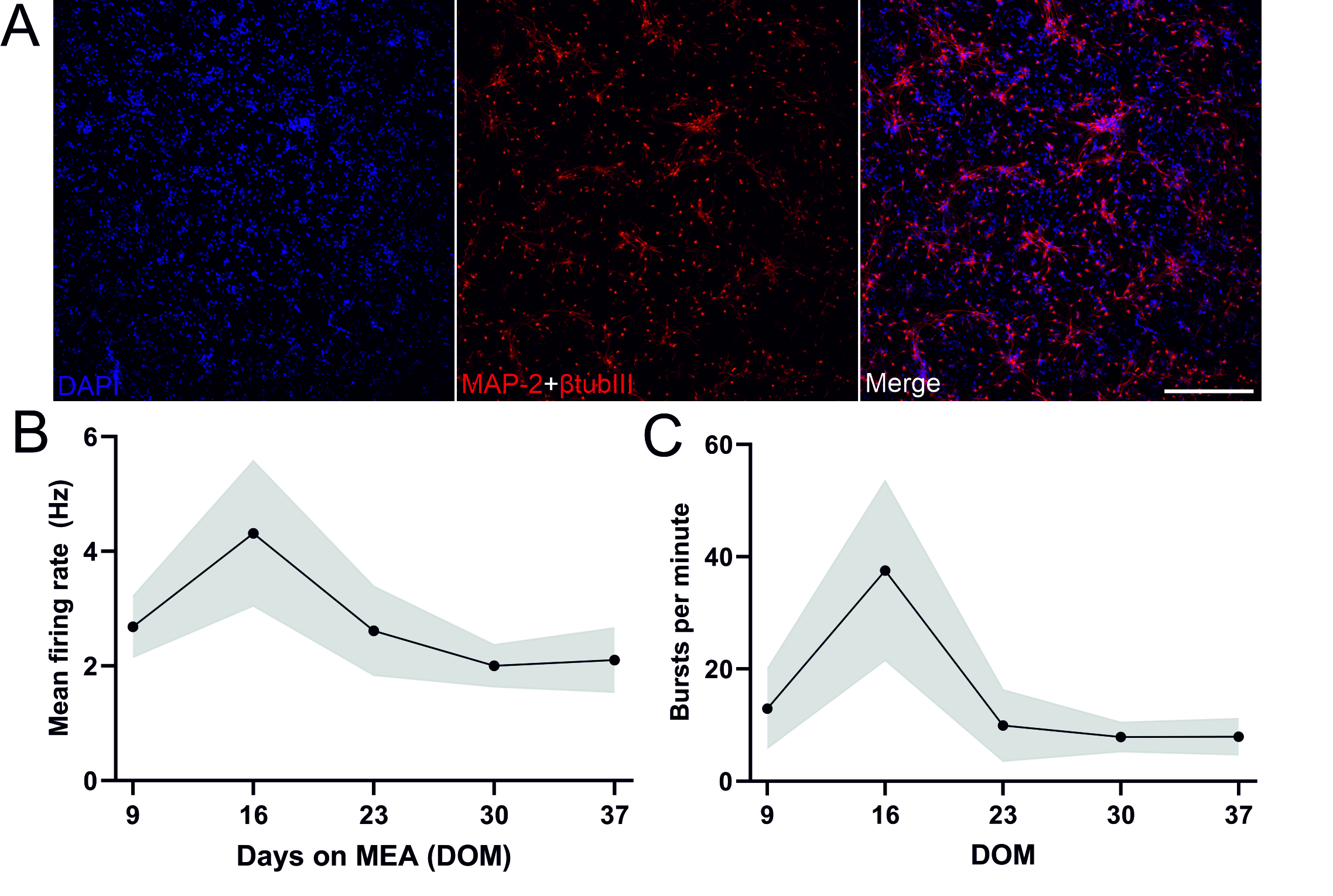


**Supplementary Figure S2**. **A** Cortical neurons (CNs) derived from TUBA1B WTC hiPSCs express MAP-2+βtub_III_ and nuclei marker DAPI. Scale bar 300 µm. **B**-**C** The development of spontaneous neuronal activity on MEA from DOM 9 to DOM 37. Parameters (**B**) mean firing rate on the active electrodes (Hz) and (**C**) mean burst rate in the burst detecting electrodes in cortical networks differentiated from TUBA1B WTC hiPSC line. The data is presented as mean + 95 % confidence interval.

**References**

1. Hyvärinen T, Hyysalo A, Kapucu FE, Aarnos L, Vinogradov A, Eglen S, et al. Functional characterization of human pluripotent stem cell-derived cortical networks differentiated on laminin-521 substrate: comparison to rat cortical cultures. Scientific Reports. 2019;9(1):17125.

2. Gelfman S, Wang Q, Lu YF, Hall D, Bostick CD, Dhindsa R, et al. (2018) meaRtools: An R package for the analysis of neuronal networks recorded on microelectrode arrays. PLoS Computational Biology. 2018;14(10):e1006506.
