# Supplementary material for: Human induced pluripotent stem cell-derived neurons and coculture conditions regulate the adipogenic differentiation and functionality of human adipose stromal/stem cells
Source: Cell Commun Signal. 2025 Nov 24;23:545. doi: 10.1186/s12964-025-02544-x (PMC12751193; doi:10.1186/s12964-025-02544-x)
Supplement: Supplementary file 8 — Supplementary Material 8. Supplementary Table 4: Performed analyses, used cell lines, cultures and media. [file 12964_2025_2544_MOESM8_ESM.docx]

**Supplementary Material 8**

**Supplementary Table 4.** Performed analyses, used cell lines, cultures and media

| **ANALYSES** | **CELL LINES** | **CULTURE + MEDIUM** |
| --- | --- | --- |
| ICC | ASC1, ASC2, ASC3 | ASC AM, ASC NM-AM |
|  | TUBA1B WTC, UTA.04511.WTs | CN NM, CN NM-AM |
|  |  | CO AM, CO NM, CO NM-AM |
| Calcium imaging | ASC2, ASC3 | ASC NM-AM |
|  | TUBA1B WTC | CN NM-AM |
|  |  | CO NM-AM |
| ELISA | ASC1, ASC2, ASC3 | ASC AM, ASC NM-AM |
|  | TUBA1B WTC, UTA.04511.WTs | CN NM-AM |
|  |  | CO NM-AM |
| FA uptake | ASC2, ASC3 | ASC NM-AM |
|  | TUBA1B WTC | CO NM-AM |
| Lipolysis assay | ASC1, ASC2, ASC3 | ASC AM, ASC NM-AM |
|  | TUBA1B WTC | CO NM-AM |
| Surface area % | ASC1, ASC2, ASC3 | ASC AM, ASC NM-AM |
|  | TUBA1B WTC | CO AM, CO NM-AM |
