# Supplementary material for: Human induced pluripotent stem cell-derived neurons and coculture conditions regulate the adipogenic differentiation and functionality of human adipose stromal/stem cells
Source: Cell Commun Signal. 2025 Nov 24;23:545. doi: 10.1186/s12964-025-02544-x (PMC12751193; doi:10.1186/s12964-025-02544-x)
Supplement: Supplementary file 9 — Supplementary Material 9. Supplementary Figure S3: ICC staining of neurons. [file 12964_2025_2544_MOESM9_ESM.docx]

**Supplementary Material 9**

**
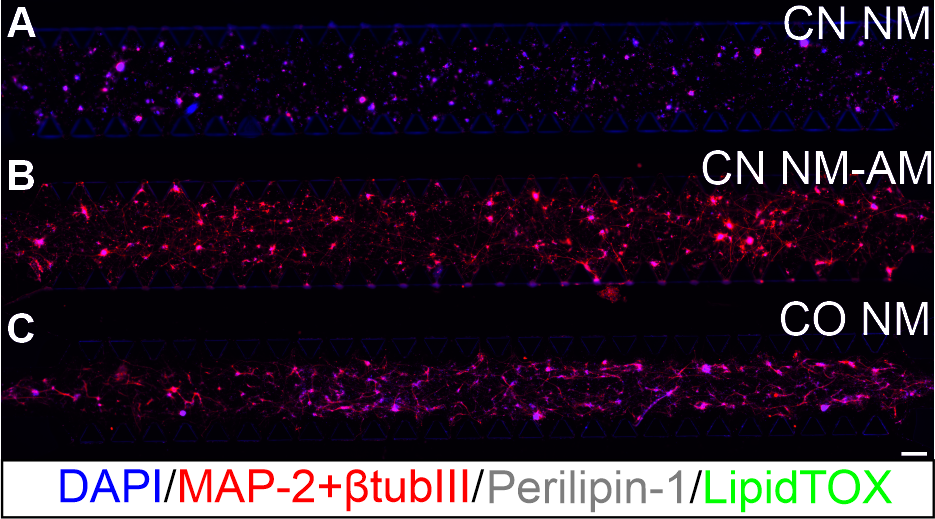
**

**Supplementary Figure S3**. **A** Cortical neurons (CNs) in neural maintenance medium (NM) express MAP2/βtub_III_ (red) but not perilipin (grey) or LipidTOX (green), similarly as CNs in neuro-adipose combination medium (NM-AM) (**B**), and coculture CNs in NM (**C**). Scale bar 300 µm.
